# Characterization of Novel *Erwinia amylovora* Jumbo Bacteriophages from *Eneladusvirus* Genus

**DOI:** 10.3390/v12121373

**Published:** 2020-11-30

**Authors:** Sang Guen Kim, Sung Bin Lee, Sib Sankar Giri, Hyoun Joong Kim, Sang Wha Kim, Jun Kwon, Jungkum Park, Eunjung Roh, Se Chang Park

**Affiliations:** 1 Laboratory of Aquatic Biomedicine, College of Veterinary Medicine and Research Institute for Veterinary Science, Seoul National University, Seoul 08826, Korea; imagine0518@snu.ac.kr (S.G.K.); lsbin1129@naver.com (S.B.L.); giribiotek@gmail.com (S.S.G.); hjoong1@nate.com (H.J.K.); kasey.kim90@gmail.com (S.W.K.); kjun1002@snu.ac.kr (J.K.); 2 Crop Protection Division, National Institute of Agriculture Sciences, Rural Development Administration, Wanju 55365, Korea; jungkuum@korea.kr (J.P.); rosalia51@korea.kr (E.R.)

**Keywords:** jumbo phage, giant phage, bacteriophage, *Eneladusvirus*, *Erwinia amylovora*, fire blight

## Abstract

Jumbo phages, which have a genome size of more than 200 kb, have recently been reported for the first time. However, limited information is available regarding their characteristics because few jumbo phages have been isolated. Therefore, in this study, we aimed to isolate and characterize other jumbo phages. We performed comparative genomic analysis of three *Erwinia* phages (pEa_SNUABM_12, pEa_SNUABM_47, and pEa_SNUABM_50), each of which had a genome size of approximately 360 kb (32.5% GC content). These phages were predicted to harbor 546, 540, and 540 open reading frames with 32, 34, and 35 tRNAs, respectively. Almost all of the genes in these phages could not be functionally annotated but showed high sequence similarity with genes encoded in *Serratia* phage BF, a member of *Eneladusvirus*. The detailed comparative and phylogenetic analyses presented in this study contribute to our understanding of the diversity and evolution of *Erwinia* phage and the genus *Eneladusvirus*.

## 1. Introduction

Bacteriophages (phages) have recently been proposed as alternative treatments to mitigate bacterial diseases [1,2,3,4,5]. Numerous novel phages have been reported since their first identification by d’Herelle in the early the 1900s. It is estimated that approximately 10^31^ to 10^32^ phages exist on our planet; however, compared with their natural host, bacteria, relatively little is known about phages [6]. In particular, despite extensive studied in the last 5 years, the total number of genome sequences available for jumbo bacteriophages, which have very large genomes (>200 kb), is only approximately around 200 [7,8,9,10,11].

Fire blight is a contagious disease in rosaceous plants, such as apples and pears, caused by *Erwinia amylovora* [1,2,3,4,5]. Since the disease seriously damages apple and pear farms, an effective alternative to antibiotics against *E. amylovora* is urgently required. Of the around 200 jumbo bacteriophage sequences currently reported, phages infecting gram-negative bacterial families in the order *Enterobacterales* account for a relatively high proportion (65 species). Most of these phages belong to the family *Myoviridae*, which has a genome size of 200–300 kb, and most species (29 species) are included in the genera *Agricanvirus, Machinavirus, Erskinevirus*, and *Derbicusvirus*, which share the same host (*Erwinia*) [4,12,13,14]. Among the dozens of jumbo phages having a genome size over 300 kb, only one phage shows infectivity towards *Erwinia* and closely related bacterial species [15]. 

One possible explanation for the evolution of the large genome is evolution (in the direction of reducing dependence on the host) as a result of competition [16,17,18]. The large genome characteristically encodes numerous tRNAs, proteins involved in regulation of metabolism, and structural proteins. In particular, tRNAs encoded by phages are thought to facilitate translation. Virion-associated RNA polymerases facilitate the independence of phages on host enzymes, and nucleus-like structural proteins form a barrier over the phage genome and interfere with the action of CRISPR-Cas, a bacterial defense system against phages [19,20,21]. The bacterial inhibition ability of jumbo phages as a result of competition has high potential for biotechnological applications, particularly phage therapy [22,23]. Incorporating jumbo phages with a variety of tools to combat bacteria into therapeutic phage libraries may provide alternative options for the treatment of diseases caused by antibiotic-resistant bacteria [24].

In this study, we aimed to improve our understanding of the evolution of jumbo bacteriophages. To this end, we focused on elucidation of the genomic characteristics of three novel jumbo bacteriophages (pEa_SNUABM_12, pEa_SNUABM_47, and pEa_SNUABM_50), which infect *E. amylovora*. Overall, our findings supported that these phages were highly similar and evolved within *Eneladusvirus*.

## 2. Material and Methods

### 2.1. Phage Isolation, Purification, and Propagation

For isolation of phages infecting *E. amylvora*, we used 220 samples (126 water samples and 94 soil samples) from the fire blight outbreak area in South Korea. Isolation was performed as previously described [25,26]. Exponentially growing bacterial cultures were mixed with the samples at a 1:1 ratio and cultured for 24 h at 27 °C to enrich the potential phages. Then, serial dilutions of the enrichment culture were dropped on the lawn of the host strain layered on the bottom agar. Samples showing inhibition spots or plaques were collected and filtered (0.45 μm) to confirm the lytic activity of phages by the conventional double layer agar (DLA) method. The plaque was cloned five times to isolate the phages.

### 2.2. Propagation, and Purification of Phages

The isolated phages were propagated using the DLA method as previously described [27]. The top agar was collected and mixed with SM buffer (100 mM NaCl, 50 mM Tris (pH 7.5), and 10 mM MgSO4) for 1 h to elute the virus particles in the agar. To remove contaminants, the sample was centrifuged and filtered (0.45 μm). Then, the phage particles were precipitated using polyethylene glycol/NaCl solution. To separate phage particles from bacterial particles and debris, the sample was treated by CsCl gradient purification method [28]. The samples with the gradient CsCl solution were ultra-centrifuged with 50,000× *g* for 2 h 30 min using Type 70 Ti Fixed-Angle Titanium Rotor (Beckman, Brea, CA, USA). The bacteriophage bands were collected and these were dialyzed with 7000 MWCO Slide-A-Lyzer ^®^ Dialysis Cassette (Thermo Scientific, Waltham, MA, USA). The purified phage solution (>10^10^ plaque forming units [PFU]/mL) was stored at 4 °C for further use.

### 2.3. Transmission Electron Microscopy

Ten microliters of purified phage solution was spotted on a copper grid for 1 min. Then, 2% phosphotungstic acid was used to stain the samples, and excess solution was removed. The grids were allowed to air dry and were then observed with a Talos L120C transmission electron microscope (FEI, Hillsboro, OR, USA) operated at 120 kV. The size of the phage particles was calculated by measuring three independent virions (*n* = 3).

### 2.4. Host Range Analysis

Several bacterial genera including *Erwinia, Escherichia, Pectobacterium, Pseudomonas*, and *Serratia* were used for host range analysis (Table 1). Serial dilutions (10^−1^ to 10^−8^) were spotted on the lawn of each bacterial species and incubated for 24 h at 27 °C. The presence of zone of inhibition or plaque on the spot was considered as susceptible strains.

### 2.5. DNA Preparation and Sequencing

The genomic DNA of phages was isolated using the conventional phenol-chloroform method [25,26]. To hydrolyze the bacterial nucleotides, 1 mL phage solution (10^10^ PFU/mL) was incubated with 10 U of DNase I and RNase A for 1 h at 37 °C. Then, the enzymes were inhibited with 50 μL ethylenediaminetetraacetic acid (0.5 M) for 15 min, followed by the addition of proteinase K for 3 h at 56 °C. The solution was mixed with phenol:chloroform:isoamylalchol (25:24:1) and centrifuged. The supernatant was precipitated with ethanol and resuspended in TE buffer. The isolated DNA was sequenced using an Illumina HiSeq (Illumina, San Diego, CA, USA) at Genotech (Daejeon, South Korea). Trimming and assembly of the reads were performed using CLC Genomic Workbench (v6.5.1). End sequence was confirmed by PCR Direct Sequencing.

### 2.6. Genome Analysis

The prediction and annotation of open reading frames (ORFs) were conducted using GenMarkS (v4.28), Prokka (v1.12), and protein BLAST with default setting [29,30]. The presence of tRNA, antimicrobial resistance, or virulence-related genes was analyzed using tRNAscan-SE (v2.0), ResFinder (v3.2), and VirulenceFinder (v2.0), respectively [31,32,33]. The genome was visualized using DNA plotter [34]. For comparative genome analysis, Gepard, Easyfig, Virus Classification and Tree Building Online Resource (VICTOR), and MEGA were adopted [35,36,37,38]. The dot plots of the genomes were obtained with default settings for Gepard, except for the whole genome dot plot, which used a word size of 12. The sequence similarity between the phages was analyzed using tBLASTx with default setting in Easyfig software. Phylogenetic analysis of the whole genomes was performed in VICTOR with the recommended settings for prokaryotic viruses, using 32 jumbo phages infecting *Erwinia* spp. and 33 jumbo phages infecting the bacteria included in order of *Enterobacterales*, and analysis of particular genes (e.g., major capsid protein, terminase, and tail sheath) was performed using the maximum likelihood method after alignment with MUSCLE implemented in MEGA X with 1000 bootstraps. The nucleotide identity of 65 jumbo phages was analyzed by EMBOSS Stretcher with default setting [39].

### 2.7. Proteome Analysis

The proteome analysis was performed as previously described [40]. The phage ghosts were prepared by freezer thaw method and separated by SDS-PAGE using 12% gel. The proteins were in gel digested with 100 ng/μL of trypsin. The proteins of bacteriophages were analyzed by nano high resolution LC-MS/MS spectrometer Q Exactive Hybrid Quadrupole-Orbitrap (Thermo Scientific, Waltham, MA, USA) equipped with Dionex U 3000 RSLC nano HPLC system.

### 2.8. Adsorption Assay and One-Step Growth Curve

The adsorption assay and one-step growth assay were performed as previously described [25]. The exponentially growing 4.0 × 10^8^ CFU/mL host strain (*E. amylovora* SNUABM_01) was co-cultured with the phages (MOI 0.001) at 27 °C. For the adsorption assay, 100 μL of samples were taken at 1, 3, 5, 7, 10, 15, 20, and 30 min and centrifuged to determine the number of un-adsorbed phages. For the one-step growth assay, phages were adsorbed for 15 min and sampled to determine the latent period and burst size for 140 min at an interval of 20 min.

### 2.9. Data Availability

The complete genome sequences of the three *Erwinia* jumbo phages (pEa_SNUABM_12, pEa_SNUABM_47, and pEa_SNUABM_50) were deposited at GenBank under the accession numbers MT939486, MT939487, and MT939488, respectively.

## 3. Results and Discussions

### 3.1. Biological Characteristics of pEa_SNUABM_12, pEa_SNUABM_47, and pEa_SNUABM_50

**Figure 1:**
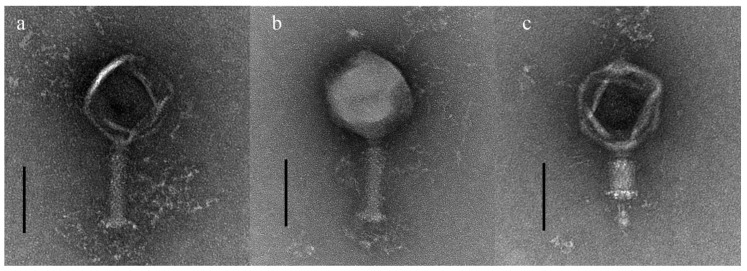
Transmission electron micrographs of *Erwinia* phages pEa_SNUABM_12 (**a**), pEa_SNUABM_47 (**b**), pEa_SNUABM_50 (**c**); the microscope was operated at 120kV. Scale bar: 100nm.

Electron microscopy analysis showed that the phages were morphologically assigned to the *Myoviridae* family (Figure 1). The phages possess a capsid 130 ± 5.9 nm in diameter (n = 5) and a contractile tail 126.7 ± 2.6 nm in length (n = 5). The host range of the jumbo *Erwinia* phages pEa_SNUABM_12, pEa_SNUABM_47, and pEa_SNUABM_50 is represented in Table 1. The jumbo phages can infect the two *Erwinia* species, *E. amylovora* and *E. pyrifoliae*, that causes blight on the rosaceous plants, and *S. marcescens*. The lysis from without was observed at the higher concentration of the spots in the *P. carotovorum*.

### 3.2. General Features of the pEa_SNUABM_12, pEa_SNUABM_47, and pEa_SNUABM_50 Genomes

The general features of the isolated phages are shown in Table 2. The general features of the isolated phages were similar to those of *Eneladusvirus* which has a 354–357 kb double-stranded DNA genome with 34.4% GC content, 531–549 ORFs, and 29–31 tRNAs (Table 2) [41]. *Erwinia* phages pEa_SNUABM_12, pEa_SNUABM_47, and pEa_SNUABM_50 possessed relatively large genomes (358,115, 355,376, and 356,948 bp, respectively), even among other jumbo phages. From the assembly results, these phages were found to have a circular double-stranded DNA for their genomes, with very low GC content (approximately 34.4%). In total, 546, 540, and 540 ORFs predicted in the genomes of pEa_SNUABM_12, pEa_SNUABM_47, and pEa_SNUABM_50, respectively, were evenly distributed in the clockwise direction. In addition, 32–35 tRNAs were identified, and no antibiotic resistance- or virulence-related genes were detected. 

### 3.3. Comparative Analysis of the pEa_SNUABM_12, pEa_SNUABM_47, and pEa_SNUABM_50 Genomes

BLAST search based on the nucleotide sequence indicated that the *Erwinia* phages pEa_SNUABM_12, pEa_SNUABM_47, and pEa_SNUABM_50 had closer relationship with RaK2-like phages, which infect *Enterobacterales*. These phages consisted of 16 species included in four genera and two unclassified species, i.e., *Asteriusvirus* (*Escherichia* phage CMSTMSU, *Escherichia* phage vB_EcoM_G17, *Escherichia* phage UB, *Escherichia* phage PBECO_4, *Escherichia* phage Ecwhy_1, *Escherichia* phage vB_EcoM_phAPEC6, *Escherichia* phage vB_Eco_slurp01, *Escherichia* phage SP27, *Escherichia* phage 121Q), *Alcyoneusvirus* (*Klebsiella* phage K64–1 and *Enterobacteria* phage vB_KleM-RaK2), *Mimasvirus* (*Cronobacter* phage vB_CSaM_GAP32), *Eneladusvirus* (*Pectobacterium* phage CBB, *Serratia* phage BF, *Yersinia* phage fHe-Yen9–03, and *Yersinia* phage fHe-Yen9–04), and unclassified (*Salmonella* phage Munch, *Salmonella* phage 7t3). The closest species of the isolated phages was *Serratia* phage BF, which had 97.9% (535 of 546 ORFs), 97.9% (529 of 540 ORFs), and 98.7% (533 of 540 ORFs) protein sequence similarity with phage pEa_SNUABM_12, pEa_SNUABM_47, and pEa_SNUABM_50, respectively (Tables S1–S3).

**Figure 2:**
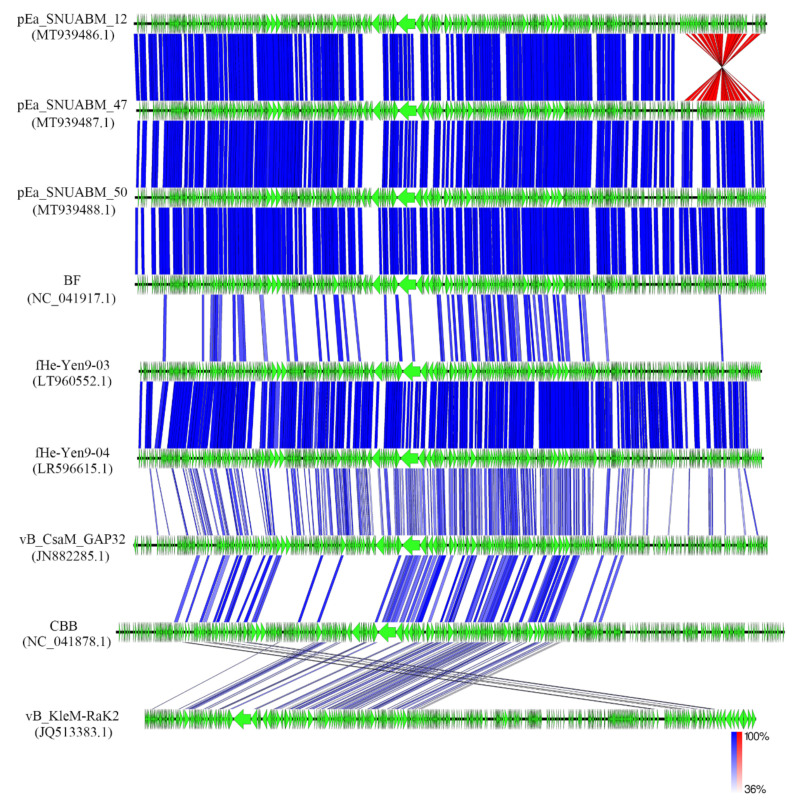
Comparative whole genome analysis of *Erwinia* phages pEa_SNUABM_12, pEa_SNUABM_47, and pEa_SNUABM_50 and related jumbo phages. The analysis was performed with tBLASTx algorithm in Easyfig.

Comparative genome analysis with RaK2-like phages showed that the genome of pEa_SNUABM_12 was arranged in opposite orientation (red) at the last region of their genome compared with the other two phages (pEa_SNUABM_47 and pEa_SNUABM_50) isolated in this study. The blue shades indicate normal orientation. They showed high sequence similarity with *Serratia* phage BF (Figure 2).

### 3.4. Phylogeneitc Analysis of the pEa_SNUABM_12, pEa_SNUABM_47, and pEa_SNUABM_50 Genomes

**Figure 3:**
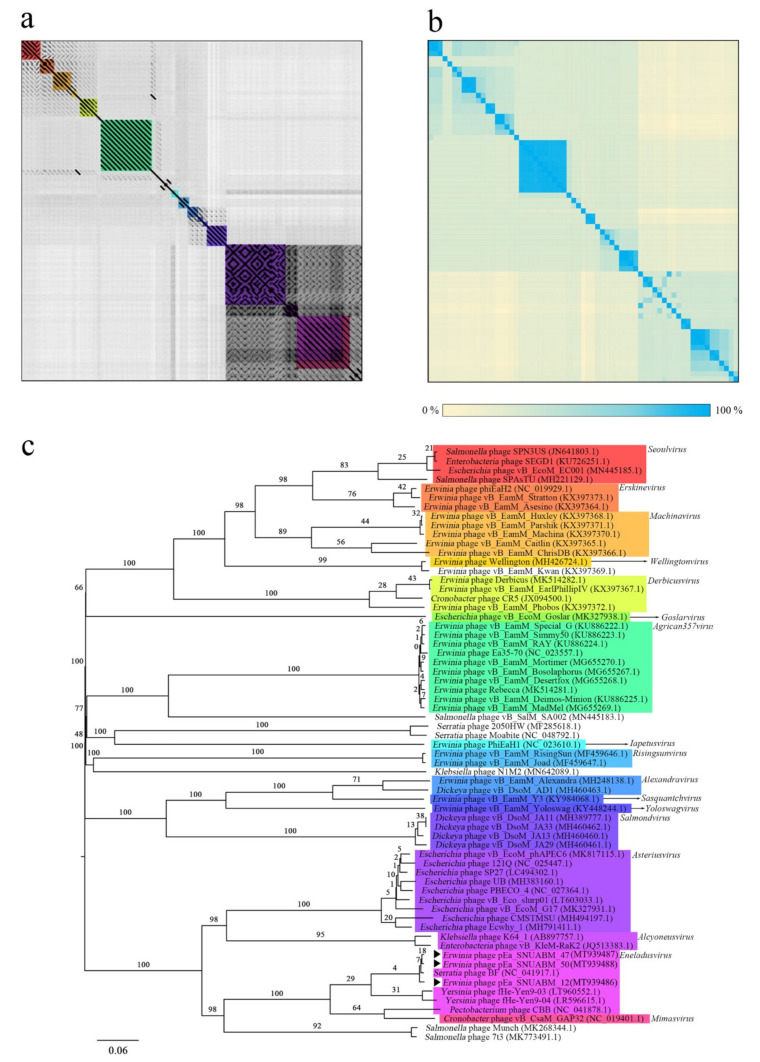
Whole genome phylogenetic analysis of *Myoviridae* jumbo phages infecting *Enterobacterales*. Dot plot (**a**) and average nucleotide identity (ANI) heat map (**b**) of 65 jumbo phages with genomes over 200 kb. The ANI value can be found in Table S4. The phylogenetic tree was created from VICTOR with default settings for prokaryotic viruses (**c**). The phages isolated in this study were indicated with black arrow (▶). *Seoulvirus* is marked with red box, *Erskinevirus* with orange box, *Machinavirus* with light orange box, *Wellingtonvirus* with yellow box, *Derbicusvirus* with yellowish green box, *Goslarvirus* with green box, *Agrican357virus* with emerald box, *Iapetusvirus* with light blue box, *Risingsunvirus* with sky-blue box, *Alexandravirus* with blue box, *Sasquantchvirus* with deep blue box, *Yoloswagvirus* with dark blue box, *Salmondvirus* with violet box, *Asteriusvirus* with purple box, *Alcyoneusvirus* with orchid pink box, *Eneladusvirus* with pink box, and *Mimasvirus* with dark pink box (**a**,**c**).

To further investigate the phylogenetic relationship of the jumbo phages pEa_SNUABM_12, pEa_SNUABM_47, and pEa_SNUABM_50, genome sequences of 65 jumbo phages that infect *Enterobacterales* (*Cronobacter, Dickeya, Erwinia, Escherichia, Klebsiella, Salmonella, Serratia*, and *Yersinia*) were evaluated. Whole-genome sequences and specific gene sequences (major capsid protein, terminase large subunit, and tail sheath protein) were analyzed using dot plots, average nucleotide identity and phylogeny. Whole-genome phylogenetic analysis using VICTOR yielded accurate classifications according to the genus of the jumbo phages. The results showed that the three isolated phages from this study formed a cluster with *Serratia* phage BF rather clustering with other *Erwinia* phages, and this cluster branched from the common ancestor with *Yersinia* phages fHe_Yen9_03 and fHe_Yen9_04, members of *Eneladusvirus* (Figure 3). The dot plot and average nucleotide identity (ANI) with whole-genome sequences supported the results of the phylogenetic tree, which showed strong clustering according to genus (Figure 3a,b). The three jumbo phages isolated from this study did not have any link with other *Erwinia* phages, but were strongly connected with members of *Eneladusvirus* (*Serratia* phage BF, *Yersinia* phages fHe_Yen9_03 and fHe_Yen9_04, and *Pectobacterium* phage CBB) and *Mimasvirus* (*Cronobacter* phage vB_CsaM_GAP32; Figure 3c).

**Figure 4:**
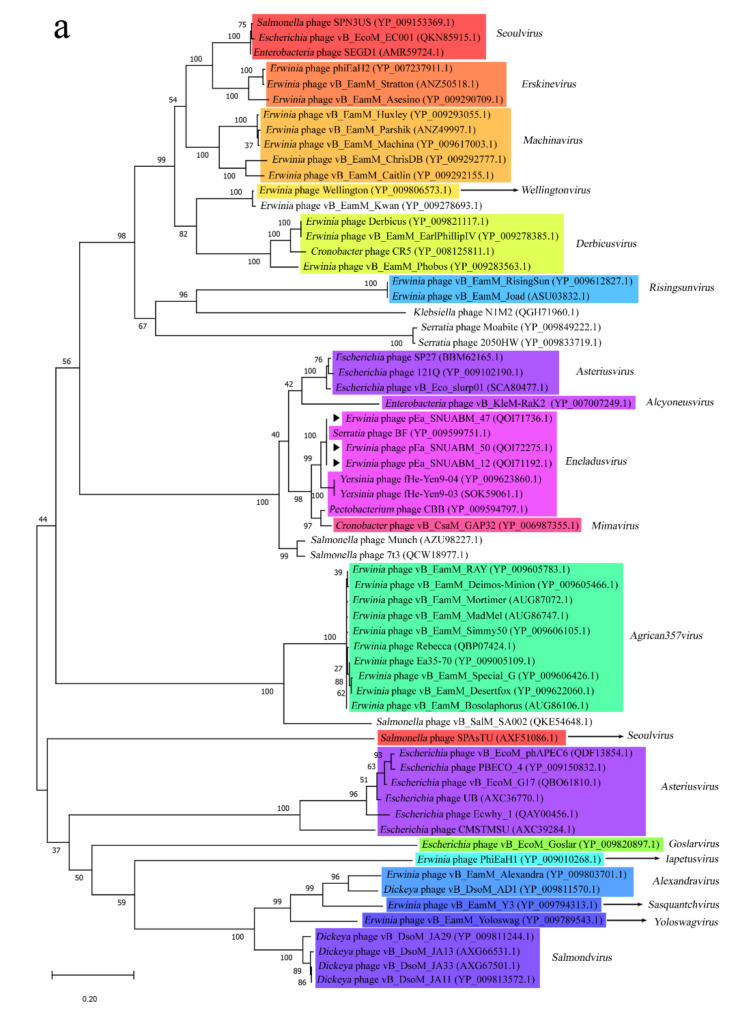

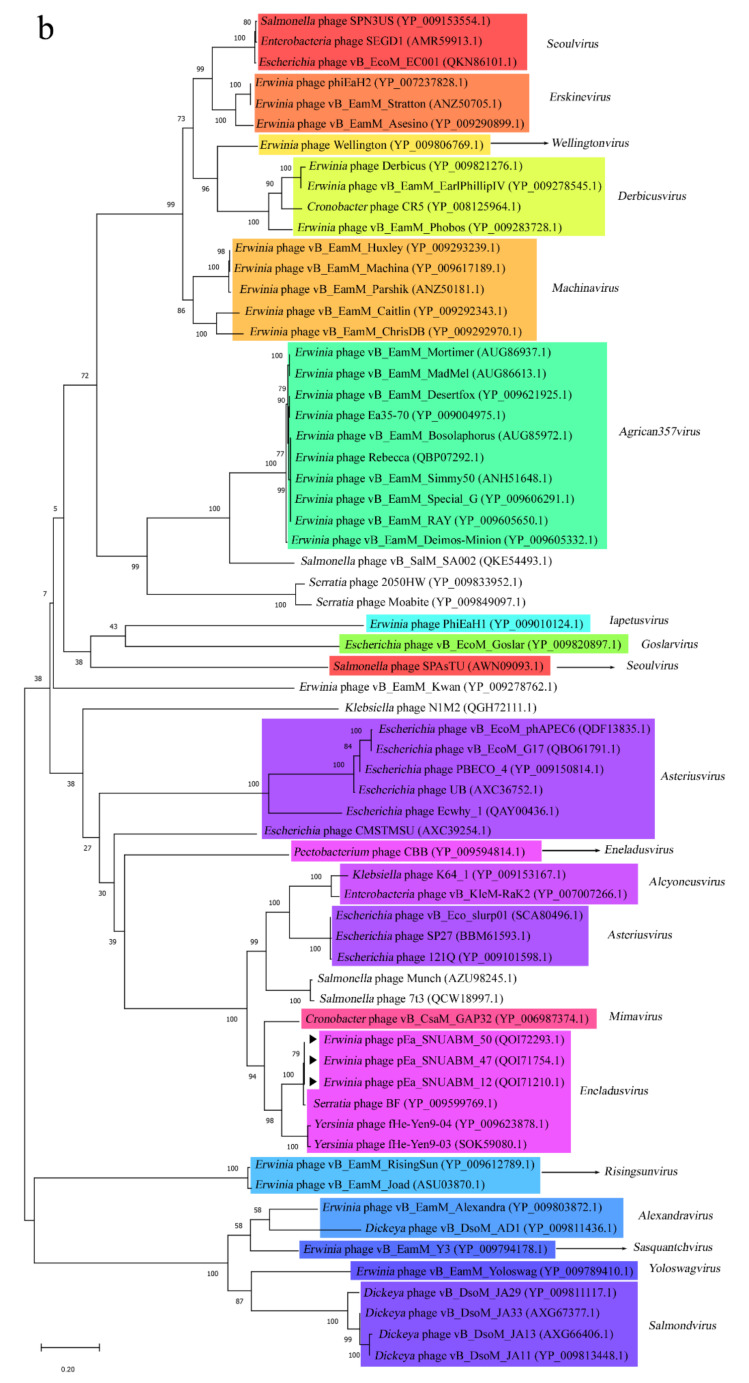

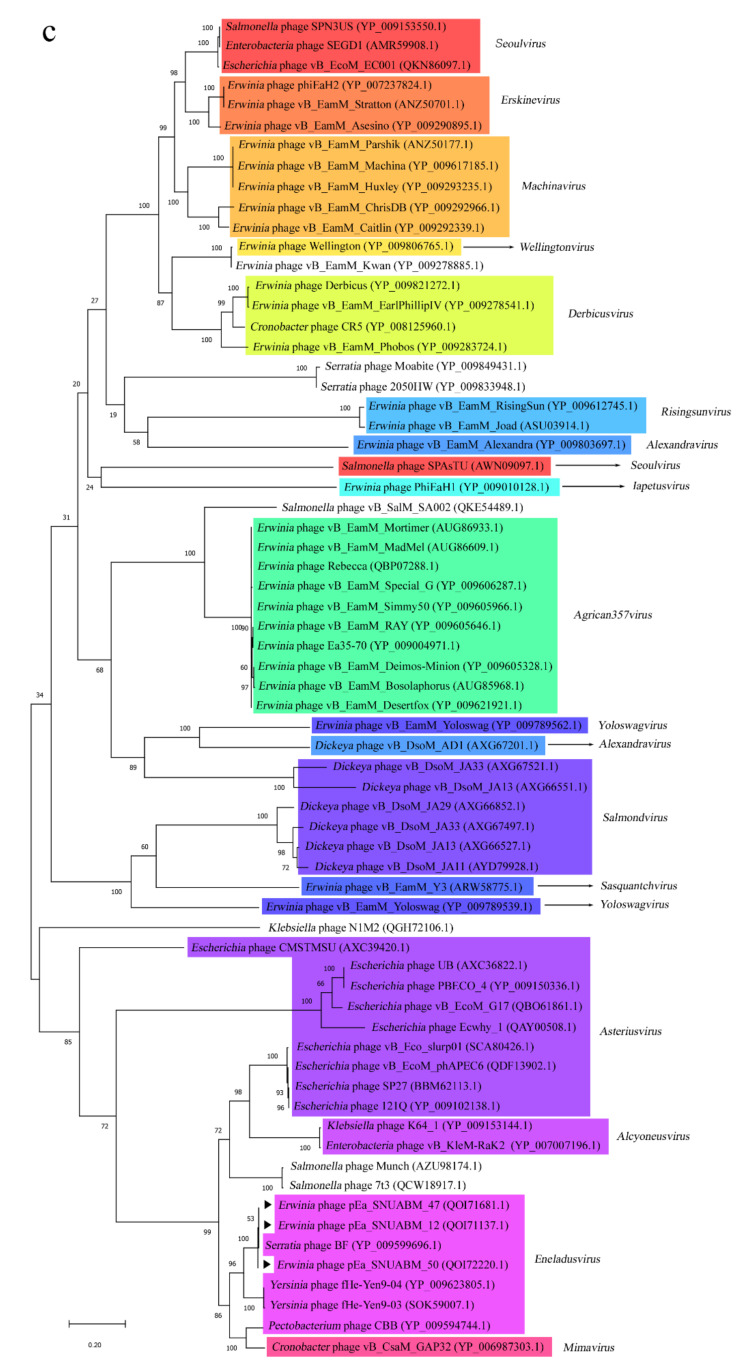
Phylogenetic analysis of *Myoviridae* jumbo phages infecting *Enterobacterales* using major capsid protein (**a**); terminase large subunit (**b**); tail sheath protein (**c**); the phylogenetic trees were created from MEGA with the maximum likelihood algorithm. The phages isolated in this study were indicated with black arrow (▶).

Phylogenetic analysis using the major capsid protein and terminase large subunit could not properly cluster the related phages, although they are commonly used as markers for phylogenetic analysis (Figure 4a,b) [42]. Thus, we used the tail sheath genes because all jumbo phages infecting *Enterobacterales* were myoviruses (Figure 4c). Classification using tail sheath proteins as a phylogenetic marker yielded phylogeny trees according to genus, as shown in the whole-genome phylogenetic tree (Figure 4c). Based on these results, we suggest that the *Erwinia* jumbo phages pEa_SNUABM_12, pEa_SNUABM_47, and pEa_SNUABM_50 may be members of *Eneladusvirus* and that the tail sheath protein could be used as a phylogenetic marker for jumbo phages infecting *Enterobacterales.*

### 3.5. Functional Analysis of the pEa_SNUABM_12, pEa_SNUABM_47, and pEa_SNUABM_50 Genomes

The phages showed similar genome arrangement, except that the last region of the pEa_SNUABM_12 genome was arranged in the opposite orientation compared with the other two phages. Furthermore, pEa_SNUABM_12 had six more predicted proteins than the other two phages, one of which was a Kelch-like protein and the others were hypothetical proteins. Among the viruses, Kelch-like proteins are isolated from poxviruses and are considered to be related to virulence against the host. Although these proteins have been reported in some jumbo phages, their functions have not yet been elucidated [43].

**Figure 5:**
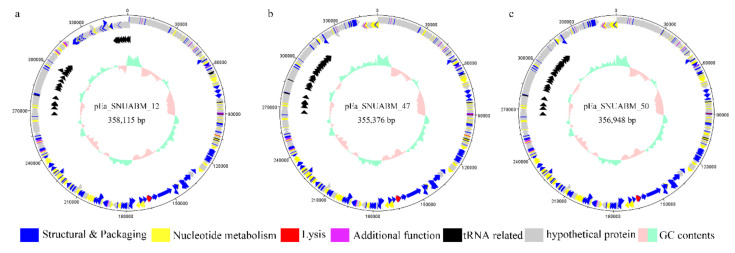
Whole genome map of *Erwinia* phages (**a**) pEa_SNUABM_12; (**b**) pEa_SNUABM_47; and (**c**) pEa_SNUABM_50. Predicted ORFs were functionally categorized and colored; Blue represents proteins with structural and packaging functions, yellow represents proteins with nucleotide metabolism functions, red represents lysis functions, purple represents proteins with additional functions, black represents tRNAs or tRNA related functions, and gray represents proteins with hypothetical functions. Scale = base pair.

Similar to other *Eneladusviruses*, more than 60% of the predicted ORFs did not have matches in any database, and their functions were unknown [44,45]. However, based on a similarity analysis in a previous study, the functions of the proteins from pEa_SNUABM_12, pEa_SNUABM_47, and pEa_SNUABM_50 could be classified into six categories: structural and packaging proteins, tRNA modification proteins and tRNAs, nucleotide metabolism-related proteins, lysis-related proteins, proteins with additional functions, and hypothetical proteins (Figure 5, Tables S1–S3).

#### 3.5.1. Mass Spectrometry 

**Figure 6:**
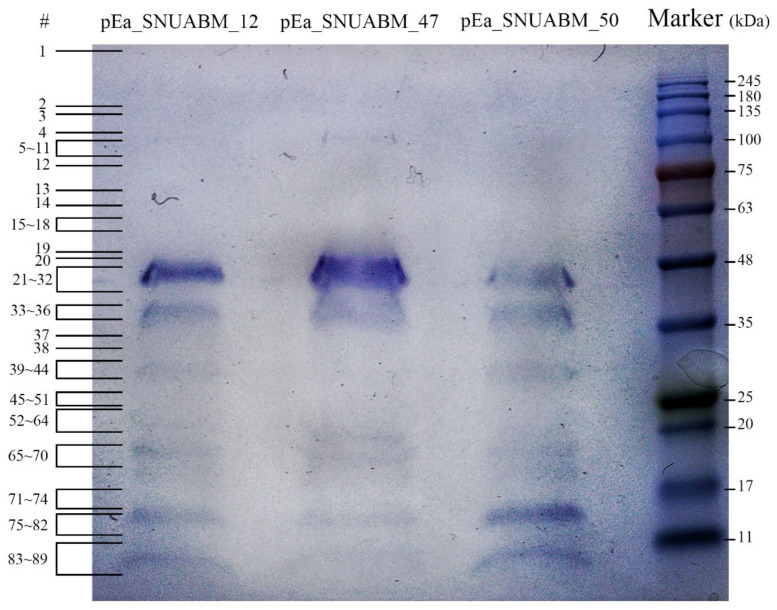
Protein analysis of phages pEa_SNUABM_12, pEa_SNUABM_47, and pEa_SNUABM_50. The number on the left side indicates the proteins identified by mass spectrometry and match with the number in the Table S5.

SDS-PAGE was performed to analyze the protein of phages pEa_SNUABM_12, pEa_SNUABM_47, and pEa_SNUABM_50 and some novel protein bands were detected by nano high resolution LC-MS/MS spectrometer. In the proteins of these phages, mass spectrometry identified 89 proteins (Figure 6) including 48 structural and packaging proteins, 30 novel hypothetical proteins, 5 proteins with nucleotide metabolism function, 2 lysis proteins, and 4 proteins with additional function (Table S5). A number of hypothetical proteins can be considered to be related with virion structure and SPFH domain- or Ig domain-containing proteins, PE-PGRS family protein, DnaJ-like protein, and acyl carrier protein were identified by LC-MS/MS spectrometry analysis besides generally known structural proteins.

#### 3.5.2. Structure and Packaging

Structural genes were predicted based on nucleotide similarity from a previous study analyzing protein function by mass spectrometry [15]. Such predicted proteins were found to have functions in capsid structure (major capsid protein, prohead core protein, head complete protein, prohead core scaffolding protein, membrane protein, and portal vertex protein), tail (major tail protein, minor tail protein, tail sheath protein, tail sheath stabilizer and completion protein, baseplate protein, baseplate hub subunits and tail lysozyme, baseplate wedge protein, and long tail fiber proximal subunit). The structure proteins of pEa_SNUABM_12, pEa_SNUABM_47, and pEa_SNUABM_50 showed close relationship with the *Serratia* phage BF, demonstrating matching of all structural proteins. For packaging, the isolated phages harbored two terminase proteins: terminase large subunit and terminase-like protein (truncated form), which is a representative feature of Rak2-like phages [15]. The HNH endonuclease and endonuclease VII were thought to be involved in completion of packaging [46].

#### 3.5.3. tRNAs

As the isolated phages had highly lower GC contents than their hosts, these phages likely utilize their numerous tRNAs to facilitate the translation of their genome [47]. Phages pEa_SNUABM_12, pEa_SNUABM_47, and pEa_SNUABM_50 encoded 32, 35, and 34 tRNAs, respectively. pEa_SNUABM_12 contained several copies of functional tRNA genes (tRNA-Ser, tRNA-Trp, tRNA-Leu, tRNA-Arg, tRNA-Pyl, tRNA-Met, tRNA-Phe, tRNA-Lys, tRNA-Glu, tRNA-Ile, tRNA-Gln, tRNA-Asp, tRNA-Pro, tRNA-His, tRNA-Tyr, tRNA-Cys, and tRNA-Ala) and two pseudo-tRNA genes (tRNA-Thr and tRNA-Leu) with two clustered areas in their genomes (269–310 kb and 358–345 kb). pEa_SNUABM_47 had two additional functional tRNA genes (tRNA-Val and tRNA-fMet) compared with pEa_SNUABM_12, and tRNAs were clustered in two areas. The total tRNA genes of pEa_SNUABM_50 were the same as those of pEa_SNUABM_47; however, the former had one additional tRNA-Val as a functional tRNA and two different pseudo-tRNA genes (tRNA-Thr and tRNA-Pro). The formation and turnover of tRNA in Rak2-like jumbo phages is regulated by tyrosyl-tRNA synthetase, tRNAHis-5′-guanylyltransferase, and peptidyl-tRNA hydrolase [15,44,45]. Our phages possessed two more genes, tRNA nucleotidyl transferase and aspartyl-tRNA amidotransferase, which may be involved in the formation of tRNAs.

#### 3.5.4. Nucleotide Metabolism

*Erwinia* phages pEa_SNUABM_12, pEa_SNUABM_47, and pEa_SNUABM_50 used nucleotide metabolism strategies similar to those of other Rak2-like phages [15,44,45]. ORFs involved in nucleotide metabolism, such as glutaredoxin, thioredoxin, thymidine kinase, CMP/dCMP or dCMP deaminase, and aerobic/anaerobic ribonucleoside diphosphate reductase subunits, were encoded in their genomes.

DNA replication mechanisms of these *Erwinia* phages are analogous to those of T4 phage that is completed via DNA polymerases, clamp loader, loading protein, single-strand DNA binding protein, primase, and helicase [48]. The DNA replication strategy of phages isolated from this study provided a “missing link” between T4-like phages and *Eneladusvirus* because they contained clamp loader protein, which is absent from Rak2-like phages and present in T4-like phages [43,44,45]. However, the loading protein was not detected in the genome, which was mainly comprised of hypothetical proteins.

Interestingly, Rak2-like phages encode DNA modification-related genes, such as DNA condensation protein, methylase, and glycosylase, which provide protection to phages against the host defense mechanism [49]. The phages isolated in this study did not harbor glycosylase; however, methylation enzymes related to adenine or cytosine were encoded in their genomes, similar to closely related *Eneladusvirus* and *Pectobacterium* phage CBB [15].

#### 3.5.5. Lysis

The lysis strategy of the *Eneladusvirus*-associated phages pEa_SNUABM_12, pEa_SNUABM_47, and pEa_SNUABM_50 depended on lysozyme, similar to other RaK2-like phages [15,44,45,50,51] and presumably spanin. The three potential lysozymes showed high sequence similarity with T4-like tail-associated glycoside hydrolase of *Eneladusvirus*, *Serratia* phage BF, *Yersinia* phage fHe-Yen9-03, and *Yersinia* phage fHe-Yen9–04, exhibiting more than 90% identity. The homologies were lower for well-known Rak2-like phages, *Klebsiella* phage vB_KleM_RaK2, *Pectobacterium* phage CBB, and *Cronobacter* phage vB_CsaM_GAP32 (78.03%, 74.76%, and 41.58% identity, respectively).

To lyse the bacterial membrane, spanin protein usually requires two components spanning the periplasmic area: i-spanin and o-spanin for the inner membrane protein and outer membrane lipoprotein signals, respectively [52]. In some cases, uni-component spanins (u-spanin) have an outer membrane lipoprotein signal at the N-terminus and transmembrane domain at the C-terminus [53]. In the genomes of our phages from *Eneladusvirus*, we identified a spanin-like protein showing 42% of homology with o-spanin of *Salmonella* phage Munch. However, as the inner part of the protein, i-spanin was not observable in their genomes. Further studies are needed to elucidate the lysis mechanism related to the spanin.

#### 3.5.6. Additional Function

SPFH domain containing protein can be found in some RaK2-like phages such as *Serratia* phage BF, *Yersinia* phages fHe-Yen9–03 and fHe-Yen9–04, and *Cronobacter* phage vB_CsaM_GAP32. This protein is highly conserved in various organisms; however, its function in viruses, particularly in bacteriophages, is poorly understood. Although the exact function has not been elucidated, this protein is known to have potential functions in protein scaffolding in prokaryotes [54].

Serine/threonine protein phosphatase and PhoH family protein are widely encoded in *Enterobacterales* jumbo phages with genome sizes of over 300 kb, such as *Alcyoneusvirus*, *Asteriusvirus*, *Eneladusvirus*, and *Mimasvirus*. These proteins are known to be associated with regulation or signal transduction in early gene expression [55]. Acyl carrier proteins (ACPs) are rarely found in the genomes of bacteriophages. In prokaryotes, ACPs are essential for the synthesis of fatty acids in many reactions [56]. In our genome studies, this protein was found to be located near the spanin-like protein which need a lipoprotein signal in its N-terminus for full function as a lysis module. This protein has been found in jumbo phages of the genus *Eneladusvirus* and in the genome of *Serratia* phage vB_Sru IME250 [57]. 

### 3.6. Adsorption Assay and One-Step Growth Curve

**Figure 7:**
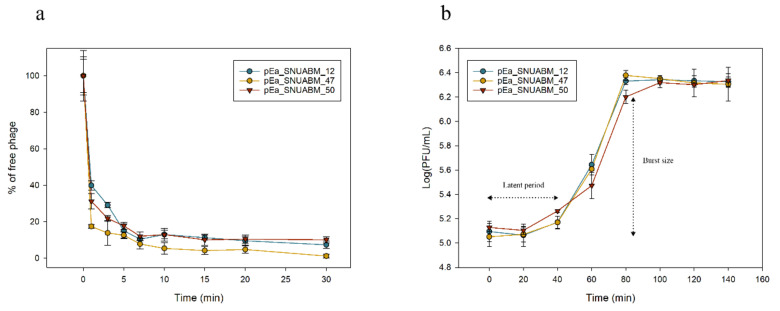
Adsorption (**a**) and one-step growth curve (**b**) of pEa_SNUABM_12, pEa_SNUABM_47, and pEa_SNUABM_50.

In adsorption assay, more than 90% of pEa_SNUABM_12, pEa_SNUABM_47, and pEa_SNUABM_50 was adsorbed into the bacteria after 15 min (Figure 7a). The experiment of one-step growth was performed to establish growth curve and burst size of each phage. The growth of phages lasted for approximately 40 min on pEa_SNUABM_12, pEa_SNUABM_47, and pEa_SNUABM_50 (Figure 7b). After the 40 min of latent period, the burst sizes of the phages were approximately 17.51 ± 1.48, 19.94 ± 3.31, and 15.51 ± 1.46 PFU per bacterial cell for pEa_SNUABM_12, pEa_SNUABM_47, and pEa_SNUABM_50 (Figure 7b). On the other hand, *Erwinia* jumbo phage Deimos-Minion (273 kbp) had latent period of approximately 3–4 h and the burst size was 4.6–4.9 PFU per bacterial cell [4]. Characterization of other jumbo phages which have similar genome size with pEa_SNUABM_12, pEa_SNUABM_47, and pEa_SNUABM_50 have not been researched, thus, further studies are required for comprehensive understand of jumbo phage biology.

## 4. Conclusions

Most jumbo phages belong to the genus *Myoviridae*; however, some *Siphoviridae* bacteriophages that infect *Caulobacter* are registered in the public depository. Jumbo phage genes mostly encode hypothetical proteins or have very low homology with other phages, suggesting that they have evolved from different niches with different evolution strategies [47]. In particular, the well-known *Pseudomonas* jumbo phage PhiKZ encodes a host-independent RNA polymerase [19]. The nucleus-like structure protein of *Serratia* jumbo phage PCH45 protects its genome from the defense system of bacteria, CRISPR-Cas, by surrounding its genome [21]. The phages isolated in this study possessed DNA condensation proteins and starvation or anaerobic environment inducible nucleotide metabolism-related proteins, similar to other phages included in *Eneladusvirus*; although these functions have not been confirmed, this is a common evolutionary strategy in the genus [15,51].

One approach for accurate estimation of evolutionary relationship is whole-genome analysis; however, aligning dozens of whole-genome sequences from phages is not possible in normal environments. In bacteriophages, particular genes (e.g., major capsid protein, and terminase large subunit) that are highly conserved in the phages are generally used as evolutionary markers for phylogenetic analysis [42]. However, for jumbo phages infecting *Enterobacterales*, terminase large subunit and major capsid are not considered appropriate because they cannot properly classify these phages at the genus level. Notably, phylogenetic analysis with VICTOR is considered a reliable method to differentiate the evolutionary status of phages for genus classification, although the number of bootstrap sets is low (~100). Phylogenic trees established using whole-genome sequences are useful for forming clusters between phages with genomes of 200–300 kb or over 300 kb or by their known genus. In addition, phylogenetic analysis using tail sheath genes could clearly classify jumbo phages infecting *Enterobacterales*. Thus, we suggest that the tail sheath protein could be used as an evolutionary marker gene for genus classification of *Myoviridae* jumbo phages infecting *Enterobacterales*.

Among jumbo phages, phages with genomes of more than 300 kb have rarely been reported; thus, little is known regarding their genes and proteins. Most of the predicted ORFs in this study were proteins with unknown functions (hypothetical proteins). Although many studies have aimed to identify clues for viral dark matter in metagenomics data from the genomes of phages [58,59,60] further studies are needed. Furthermore, the three isolated phages from this study formed a cluster with *Serratia* phage BF and this cluster branched from the common ancestor with *Yersinia* phage fHe-Yen9–03, and fHe-Yen9–04. As bacteriophages are generally species-specific, it is interesting that the three phages (pEa_SNUABM_12, pEa_SNUABM_47, and pEa_SNUABM_50) which showed high genomic similarity with *Serratia* phage BF could infect the *S. marcescens* strains. Although we could not examine the infectivity of them on all the closely related bacterial strains including *Erwinia, Pectobacterium, Cronobacter, Dickeya, Yersinia,* and *Serratia*, the phages showed intergenus infection potential like other *Eneladusvirus* [15,45]. However, the similarity of the basic characterization of these *Eneladusviruses* such as adsorption assay or one-step growth curve has not been revealed yet. The characteristics and genome sequences of *Erwinia* jumbo phages identified in this study are expected to contribute to uncovering the functions of these unknown genes and viral dark matter in the future.

## Figures and Tables

**Table 1:** Host range of *Erwinia* phages pEa_SNUABM_12, pEa_SNUABM_47, and pEa_SNUABM_50.

Bacterial Strain	Biological Origin	Host Range ^a^		
		pEa_12	pEa_47	pEa_50
*Erwinia amylovora*				
SNUABM_01 ^b^	Apple	+	+	+
SNUABM_02	Pear	+	+	+
*Erwinia pyrifoliae*				
KACC13945	Apple	+	+	+
KACC13946	Apple	+	+	+
KACC13948	Apple	+	+	+
KACC13949	Apple	+	+	+
KACC13952	Apple	+	+	+
*Erwinia rhapontici*				
KACC14080	Pear	-	-	-
*Erwinia* sp.				
KACC10370	N/A	-	-	-
KACC10403	N/A	+	+	+
*Pectobacterium carotovorum*				
KACC17004	Cabbage	-	LO	LO
KACC18645	Chinese cabbagge	-	LO	LO
KACC21701	Chinese cabbagge	LO	LO	LO
*Pseudomonas aeruginosa*				
KCCM40395	N/A	-	-	-
*Escherichia coli*				
KCTC2571	Human feces	-	-	-
*Serratia marcescens*				
KACC10502	Tropical greenhouse	+	+	+
KACC11961	Pond water	+	+	+

^a^, +, -, and LO represents susceptible, insusceptible, and lysis from without, respectively. ^b^ the indicator strain used for the isolation of the phages.

**Table 2:** General genomic features of *Erwinia* jumbo phages pEa_SNUABM_12, pEa_SNUABM_47, and pEa_SNUABM_50.

	Size (bp)	ORFs	tRNAs	GC Content (%)	DNA Circularity	Accession Number
pEa_SNUABM_12	358,115	546	32	34.41	Circular	MT939486.1
pEa_SNUABM_47	355,376	540	35	34.48	Circular	MT939487.1
pEa_SNUABM_50	356,948	540	34	34.45	Circular	MT939488.1

## Supplementary Materials

The following are available online at https://www.mdpi.com/1999-4915/12/12/1373/s1, Table S1: Functional classification of ORFs in *Erwinia* phage pEa_SNUABM_12; Table S2: Functional classification of ORFs in *Erwinia* phage pEa_SNUABM_47; Table S3: Functional classification of ORFs in *Erwinia* phage pEa_SNUABM_50; Table S4: Analysis of average nucleotide identity of 65 jumbo phages infecting *Enterobacterales*; Table S5: Profiles of the LC-MS/MS detected proteins of the *E. amylovora* phage pEa_SNUABM_12, pEa_SNUABM_47, and pEa_SNUABM_50.
