# Supplementary material for: Characterization of Novel Erwinia amylovora Jumbo Bacteriophages from Eneladusvirus Genus
Source: Viruses. 2020 Nov 30;12(12):1373. doi: 10.3390/v12121373 (PMC7760394; doi:10.3390/v12121373)
Supplement: Supplementary file 1 [file viruses-12-01373-s001.zip › Supplementary Table S1.docx]

**Table S2.** Functional classification of ORFs in *Erwinia* phage pEa_SNUABM_12.

| **Group** | **Locus tag** | **Encoded protein** | **Related organism** | **Query cover**  **(%)** | **Identiy**  **(%)** |
| --- | --- | --- | --- | --- | --- |
| Structure & packaging | pEa_SNUABM12_00002 | Putative endonuclease | *Serratia* phage BF | 100 | 99.55 |
| Structure & packaging | pEa_SNUABM12_00020 | putative membrane protein | *Serratia* phage BF | 100 | 98.77 |
| Additional function | pEa_SNUABM12_00022 | SPFH domain containing protein | *Serratia* phage BF | 100 | 100 |
| Structure & packaging | pEa_SNUABM12_00027 | putative structural protein | *Serratia* phage BF | 100 | 99.35 |
| Structure & packaging | pEa_SNUABM12_00059 | putative membrane protein | *Serratia* phage BF | 100 | 100 |
| Nucleotide metabolism | pEa_SNUABM12_00063 | putative DNA N-6-adenine-methyltransferase | *Serratia* phage BF | 100 | 100 |
| Nucleotide metabolism | pEa_SNUABM12_00064 | putative cytitidyltransferase | *Serratia* phage BF | 97 | 100 |
| Nucleotide metabolism | pEa_SNUABM12_00072 | putative RNA ligase, T4 RnlA family | *Serratia* phage BF | 100 | 99.74 |
| Nucleotide metabolism | pEa_SNUABM12_00076 | putative polynucleotide 5'-kinase and 3'-phosphatase | *Serratia* phage BF | 100 | 96.75 |
| Structure & packaging | pEa_SNUABM12_00080 | putative membrane protein | *Serratia* phage BF | 100 | 100 |
| Structure & packaging | pEa_SNUABM12_00083 | putative membrane protein | *Serratia* phage BF | 100 | 100 |
| Additional function | pEa_SNUABM12_00090 | putative serine/threonine-protein phosphatase | *Serratia* phage BF | 100 | 100 |
| Nucleotide metabolism | pEa_SNUABM12_00093 | putative DNA ligase | *Serratia* phage BF | 100 | 99.58 |
| Additional function | pEa_SNUABM12_00095 | putative serine/threonine-protein phosphatase | *Serratia* phage BF | 100 | 99.25 |
| Structure & packaging | pEa_SNUABM12_00097 | putative ATP-dependent Clp protease proteolytic subunit | *Serratia* phage BF | 100 | 100 |
| Structure & packaging | pEa_SNUABM12_00102 | putative structural protein | *Serratia* phage BF | 100 | 100 |
| Nucleotide metabolism | pEa_SNUABM12_00103 | putative CMP/dCMP deaminase | *Serratia* phage BF | 100 | 99.36 |
| Structure & packaging | pEa_SNUABM12_00105 | putative major tail protein | *Serratia* phage BF | 100 | 99.34 |
| Nucleotide metabolism | pEa_SNUABM12_00106 | putative bifunctional nicotinamide mononucleotide adenylyltransferase/ADP-ribose pyrophosphatase | *Serratia* phage BF | 100 | 100 |
| Nucleotide metabolism | pEa_SNUABM12_00108 | putative nicotinamide phosphoribosyltransferase | *Serratia* phage BF | 100 | 99.8 |
| Structure & packaging | pEa_SNUABM12_00109 | putative membrane protein | *Serratia* phage BF | 100 | 100 |
| Structure & packaging | pEa_SNUABM12_00110 | putative structural protein | *Serratia* phage BF | 100 | 98.65 |
| Nucleotide metabolism | pEa_SNUABM12_00112 | putative Sir2-like protein, NAD-dependent protein deacetylase | *Serratia* phage BF | 100 | 100 |
| Nucleotide metabolism | pEa_SNUABM12_00113 | putative nudix hydrolase | *Serratia* phage BF | 100 | 100 |
| Structure & packaging | pEa_SNUABM12_00114 | putative membrane protein | *Serratia* phage BF | 100 | 100 |
| Additional function | pEa_SNUABM12_00115 | putative PhoH family protein | *Serratia* phage BF | 100 | 100 |
| Structure & packaging | pEa_SNUABM12_00116 | putative prohead core scaffolding protein | *Serratia* phage BF | 100 | 99.35 |
| Structure & packaging | pEa_SNUABM12_00119 | putative membrane protein | *Serratia* phage BF | 100 | 100 |
| Structure & packaging | pEa_SNUABM12_00120 | putative membrane protein | *Serratia* phage BF | 100 | 100 |
| tRNA related | pEa_SNUABM12_00124 | putative tyrosyl-tRNA synthetase | *Serratia* phage BF | 100 | 100 |
| Nucleotide metabolism | pEa_SNUABM12_00128 | putative adenine-specific DNA methylase | *Serratia* phage BF | 100 | 100 |
| Nucleotide metabolism | pEa_SNUABM12_00130 | putative dCMP deaminase | *Serratia* phage BF | 100 | 99.53 |
| Nucleotide metabolism | pEa_SNUABM12_00131 | putative dCMP deaminase | *Serratia* phage BF | 100 | 100 |
| Nucleotide metabolism | pEa_SNUABM12_00132 | putative AAA domain-containing ATPase | *Serratia* phage BF | 100 | 100 |
| Nucleotide metabolism | pEa_SNUABM12_00133 | putative anaerobic NTP reductase large subunit | *Serratia* phage BF | 100 | 99.84 |
| Nucleotide metabolism | pEa_SNUABM12_00137 | putative Pyruvate formate-lyase | *Serratia* phage BF | 100 | 100 |
| Nucleotide metabolism | pEa_SNUABM12_00139 | putative anaerobic ribonucleoside-triphosphate reductase activating protein | *Serratia* phage BF | 100 | 99.36 |
| Structure & packaging | pEa_SNUABM12_00140 | putative minor tail protein | *Serratia* phage BF | 100 | 98.31 |
| Structure & packaging | pEa_SNUABM12_00141 | putative structural protein | *Serratia* phage BF | 100 | 98.67 |
| Structure & packaging | pEa_SNUABM12_00142 | putative structural protein | *Serratia* phage BF | 100 | 99.67 |
| tRNA related | pEa_SNUABM12_00146 | putative tRNAHis-5'-guanylyltransferase | *Serratia* phage BF | 100 | 99.61 |
| Nucleotide metabolism | pEa_SNUABM12_00148 | putative NrdA protein | *Serratia* phage BF | 98 | 98.99 |
| Nucleotide metabolism | pEa_SNUABM12_00151 | putative site specific DNA methyltransferase | *Serratia* phage BF | 100 | 98.95 |
| Nucleotide metabolism | pEa_SNUABM12_00153 | putative nucleotidyltransferase | *Serratia* phage BF | 100 | 100 |
| Additional function | pEa_SNUABM12_00158 | putative acyl carrier protein | *Serratia* phage BF | 100 | 100 |
| Structure & packaging | pEa_SNUABM12_00159 | putative membrane protein | *Serratia* phage BF | 100 | 100 |
| Lysis | pEa_SNUABM12_00160 | putative o-spannin | *Serratia* phage BF | 100 | 99.03 |
| Structure & packaging | pEa_SNUABM12_00161 | putative structural protein | *Serratia* phage BF | 100 | 99.22 |
| Nucleotide metabolism | pEa_SNUABM12_00164 | putative starvation-inducible DNA-binding protein | *Serratia* phage BF | 100 | 100 |
| Nucleotide metabolism | pEa_SNUABM12_00168 | putative GTP cyclohydrolase | *Serratia* phage BF | 100 | 100 |
| Nucleotide metabolism | pEa_SNUABM12_00170 | putative thymidine kinase | *Serratia* phage BF | 100 | 100 |
| Structure & packaging | pEa_SNUABM12_00171 | putative tail protein | *Serratia* phage BF | 100 | 100 |
| Structure & packaging | pEa_SNUABM12_00172 | putative membrane protein | *Serratia* phage BF | 100 | 100 |
| Structure & packaging | pEa_SNUABM12_00175 | putative membrane protein | *Serratia* phage BF | 100 | 86.36 |
| Nucleotide metabolism | pEa_SNUABM12_00177 | putative RNA ligase | *Serratia* phage BF | 100 | 99.76 |
| Structure & packaging | pEa_SNUABM12_00179 | putative structural protein | *Serratia* phage BF | 100 | 100 |
| Structure & packaging | pEa_SNUABM12_00181 | putative membrane protein | *Serratia* phage BF | 100 | 95.65 |
| Lysis | pEa_SNUABM12_00184 | putative lysozyme | *Serratia* phage BF | 100 | 100 |
| Nucleotide metabolism | pEa_SNUABM12_00185 | putative sigma 54 modulation protein/ribosomal protein | *Serratia* phage BF | 100 | 100 |
| tRNA related | pEa_SNUABM12_00187 | putative tRNA nucleotidyl transferase | *Serratia* phage BF | 100 | 100 |
| Nucleotide metabolism | pEa_SNUABM12_00188 | putative nudix hydrolase | *Serratia* phage BF | 100 | 100 |
| Nucleotide metabolism | pEa_SNUABM12_00189 | putative glutaredoxin | *Serratia* phage BF | 100 | 100 |
| tRNA related | pEa_SNUABM12_00190 | putative aspartyl-tRNA amidotransferase | *Serratia* phage BF | 100 | 100 |
| Nucleotide metabolism | pEa_SNUABM12_00193 | putative Appr-1-p processing enzyme | *Serratia* phage BF | 100 | 100 |
| Structure & packaging | pEa_SNUABM12_00196 | putative neck protein | *Serratia* phage BF | 100 | 100 |
| Nucleotide metabolism | pEa_SNUABM12_00198 | putative deoxynucleotide monophosphate kinase | *Serratia* phage BF | 100 | 99.12 |
| Structure & packaging | pEa_SNUABM12_00199 | putative tail sheath protein | *Serratia* phage BF | 100 | 99.89 |
| Structure & packaging | pEa_SNUABM12_00200 | putative structural protein | *Yersinia* phage fHe-Yen9-04 | 100 | 93.45 |
| Structure & packaging | pEa_SNUABM12_00201 | putative structural protein | *Serratia* phage BF | 100 | 100 |
| Structure & packaging | pEa_SNUABM12_00202 | putative structural protein | *Serratia* phage BF | 100 | 100 |
| Structure & packaging | pEa_SNUABM12_00203 | putative head completion protein | *Serratia* phage BF | 100 | 100 |
| Structure & packaging | pEa_SNUABM12_00205 | putative structural protein | *Serratia* phage BF | 100 | 99.45 |
| Structure & packaging | pEa_SNUABM12_00206 | putative structural protein | *Serratia* phage BF | 100 | 100 |
| Structure & packaging | pEa_SNUABM12_00207 | putative structural protein | *Serratia* phage BF | 100 | 100 |
| Structure & packaging | pEa_SNUABM12_00208 | putative structural protein | *Serratia* phage BF | 100 | 100 |
| Structure & packaging | pEa_SNUABM12_00209 | putative structural protein | *Serratia* phage BF | 100 | 100 |
| Nucleotide metabolism | pEa_SNUABM12_00211 | putative ATPase | *Serratia* phage BF | 100 | 100 |
| Nucleotide metabolism | pEa_SNUABM12_00218 | putative thymidylate synthase | *Serratia* phage BF | 100 | 100 |
| Structure & packaging | pEa_SNUABM12_00219 | putative structural protein | *Serratia* phage BF | 100 | 100 |
| Structure & packaging | pEa_SNUABM12_00223 | putative structural protein | *Serratia* phage BF | 100 | 100 |
| Structure & packaging | pEa_SNUABM12_00224 | putative long tail fiber proximal subunit | *Serratia* phage BF | 100 | 99.33 |
| Structure & packaging | pEa_SNUABM12_00225 | putative structural protein | *Serratia* phage BF | 100 | 100 |
| Structure & packaging | pEa_SNUABM12_00226 | putative structural protein | *Serratia* phage BF | 100 | 100 |
| Structure & packaging | pEa_SNUABM12_00227 | putative structural protein | *Serratia* phage BF | 100 | 100 |
| Structure & packaging | pEa_SNUABM12_00228 | putative structural protein | *Serratia* phage BF | 100 | 100 |
| Structure & packaging | pEa_SNUABM12_00229 | putative structural protein | *Serratia* phage BF | 100 | 100 |
| Nucleotide metabolism | pEa_SNUABM12_00230 | putative NUDIX hydrolase family protein | *Serratia* phage BF | 100 | 100 |
| Structure & packaging | pEa_SNUABM12_00231 | putative tail sheath stabilizer and completion protein | *Serratia* phage BF | 100 | 99.78 |
| Structure & packaging | pEa_SNUABM12_00232 | putative structural protein | *Serratia* phage BF | 100 | 100 |
| Structure & packaging | pEa_SNUABM12_00233 | putative ATP-dependent Clp protease ATP-binding subunit clpA | *Serratia* phage BF | 100 | 99.74 |
| Structure & packaging | pEa_SNUABM12_00234 | putaive structural protein | *Serratia* phage BF | 100 | 99.91 |
| Structure & packaging | pEa_SNUABM12_00235 | putative baseplate wedge | *Serratia* phage BF | 100 | 100 |
| Structure & packaging | pEa_SNUABM12_00236 | putative base plate protein | *Serratia* phage BF | 100 | 100 |
| Lysis | pEa_SNUABM12_00237 | putative baseplate hub subunit and tail lysozyme | *Serratia* phage BF | 100 | 99.38 |
| Lysis | pEa_SNUABM12_00238 | putative T4-like phage baseplate hub and tail lysozyme | *Serratia* phage BF | 100 | 100 |
| Structure & packaging | pEa_SNUABM12_00239 | putative structural protein | *Serratia* phage BF | 100 | 100 |
| Structure & packaging | pEa_SNUABM12_00240 | putative baseplate wedge protein | *Serratia* phage BF | 100 | 100 |
| Structure & packaging | pEa_SNUABM12_00241 | putative structural protein | *Serratia* phage BF | 100 | 100 |
| Nucleotide metabolism | pEa_SNUABM12_00242 | putative RNA sigma factor for late transcription | *Serratia* phage BF | 100 | 100 |
| Nucleotide metabolism | pEa_SNUABM12_00243 | putative endonuclease subunit | *Serratia* phage BF | 100 | 100 |
| Nucleotide metabolism | pEa_SNUABM12_00244 | putative endonuclease subunit | *Serratia* phage BF | 100 | 99.86 |
| Structure & packaging | pEa_SNUABM12_00245 | putative EndoVII packaging and recombination endonuclease | *Serratia* phage BF | 100 | 100 |
| Structure & packaging | pEa_SNUABM12_00247 | putative baseplate hub subunit | *Serratia* phage BF | 100 | 100 |
| Structure & packaging | pEa_SNUABM12_00248 | putative tape measure protein | *Serratia* phage BF | 100 | 99.58 |
| Structure & packaging | pEa_SNUABM12_00249 | putative portal vertex protein | *Serratia* phage BF | 100 | 100 |
| Structure & packaging | pEa_SNUABM12_00251 | putative structural protein | *Serratia* phage BF | 100 | 98.98 |
| Structure & packaging | pEa_SNUABM12_00252 | putative prohead core protein | *Serratia* phage BF | 100 | 100 |
| Structure & packaging | pEa_SNUABM12_00253 | putative scaffolding protein | *Serratia* phage BF | 100 | 100 |
| Structure & packaging | pEa_SNUABM12_00254 | putative precursor of major capsid protein/ putative major capsid protein | *Serratia* phage BF | 100 | 100 |
| Nucleotide metabolism | pEa_SNUABM12_00256 | putative GIY-YIG nuclease family protein | *Serratia* phage BF | 100 | 100 |
| Structure & packaging | pEa_SNUABM12_00257 | putative tail fiber protein | *Serratia* phage BF | 100 | 100 |
| Nucleotide metabolism | pEa_SNUABM12_00260 | putative DNA polymerase | *Serratia* phage BF | 100 | 100 |
| Additional function | pEa_SNUABM12_00263 | putative serine/threonine protein phosphatase | *Serratia* phage BF | 100 | 100 |
| Additional function | pEa_SNUABM12_00264 | putative type I antifreeze protein | *Serratia* phage BF | 100 | 100 |
| Structure & packaging | pEa_SNUABM12_00265 | putative co-chaperonin GroES | *Serratia* phage BF | 100 | 98.95 |
| Structure & packaging | pEa_SNUABM12_00266 | putative structural protein | *Serratia* phage BF | 100 | 100 |
| Structure & packaging | pEa_SNUABM12_00267 | putative structural protein | *Serratia* phage BF | 100 | 100 |
| Structure & packaging | pEa_SNUABM12_00268 | putative structural protein | *Serratia* phage BF | 100 | 100 |
| Nucleotide metabolism | pEa_SNUABM12_00269 | putative RNaseH ribonuclease | *Serratia* phage BF | 100 | 100 |
| Structure & packaging | pEa_SNUABM12_00271 | putative terminase like protein | *Serratia* phage BF | 100 | 99.58 |
| Structure & packaging | pEa_SNUABM12_00272 | putative terminase large subunit | *Serratia* phage BF | 100 | 100 |
| Structure & packaging | pEa_SNUABM12_00273 | putative structural protein | *Serratia* phage BF | 100 | 99.77 |
| Nucleotide metabolism | pEa_SNUABM12_00274 | putative ssDNA binding protein | *Serratia* phage BF | 100 | 100 |
| Nucleotide metabolism | pEa_SNUABM12_00275 | putative UvsX protein | *Serratia* phage BF | 100 | 100 |
| Nucleotide metabolism | pEa_SNUABM12_00276 | putative UvsY portein | *Serratia* phage BF | 100 | 100 |
| Nucleotide metabolism | pEa_SNUABM12_00277 | putative DNA polymerase III epsilon subunit | *Serratia* phage BF | 100 | 99.66 |
| Nucleotide metabolism | pEa_SNUABM12_00278 | putative RNA-DNA + DNA-DNA helicase | *Serratia* phage BF | 100 | 100 |
| Structure & packaging | pEa_SNUABM12_00283 | putative structural protein | *Serratia* phage BF | 100 | 100 |
| Structure & packaging | pEa_SNUABM12_00284 | putative membrane protein | *Serratia* phage BF | 100 | 100 |
| Structure & packaging | pEa_SNUABM12_00286 | putative structural protein | *Serratia* phage BF | 100 | 100 |
| Nucleotide metabolism | pEa_SNUABM12_00287 | putative DNA primase subunit | *Serratia* phage BF | 100 | 100 |
| Nucleotide metabolism | pEa_SNUABM12_00288 | putative DNA primase-helicase | *Serratia* phage BF | 100 | 100 |
| Structure & packaging | pEa_SNUABM12_00292 | putative structural protein | *Serratia* phage BF | 100 | 99.67 |
| Structure & packaging | pEa_SNUABM12_00293 | putative structural protein | *Serratia* phage BF | 100 | 100 |
| Structure & packaging | pEa_SNUABM12_00295 | putative structural protein | *Serratia* phage BF | 100 | 99.54 |
| Structure & packaging | pEa_SNUABM12_00296 | putative structural protein | *Serratia* phage BF | 100 | 100 |
| Nucleotide metabolism | pEa_SNUABM12_00297 | putative restriction endonuclease type II like-protein | *Serratia* phage BF | 100 | 100 |
| Structure & packaging | pEa_SNUABM12_00299 | putative structural protein | *Serratia* phage BF | 100 | 100 |
| Nucleotide metabolism | pEa_SNUABM12_00300 | putative aerobic ribonucleotide-diphosphate reductase alpha subunit | *Serratia* phage BF | 100 | 99.87 |
| Nucleotide metabolism | pEa_SNUABM12_00301 | putative aerobic ribonucleotide-diphosphate reductase beta subunit | *Serratia* phage BF | 100 | 100 |
| Structure & packaging | pEa_SNUABM12_00302 | putative membrane protein | *Serratia* phage BF | 100 | 100 |
| Structure & packaging | pEa_SNUABM12_00304 | putative structural protein | *Serratia* phage BF | 100 | 100 |
| Nucleotide metabolism | pEa_SNUABM12_00305 | putative nucleotide pyrophosphohydrolase | *Serratia* phage BF | 100 | 99.57 |
| Structure & packaging | pEa_SNUABM12_00307 | putative structural protein | *Serratia* phage BF | 100 | 100 |
| Structure & packaging | pEa_SNUABM12_00308 | putative membrane protein | *Serratia* phage BF | 100 | 100 |
| Nucleotide metabolism | pEa_SNUABM12_00309 | putative dihydrofolate reductase | *Serratia* phage BF | 100 | 99.43 |
| Nucleotide metabolism | pEa_SNUABM12_00310 | putative ribonuclease H | *Serratia* phage BF | 100 | 100 |
| Nucleotide metabolism | pEa_SNUABM12_00311 | putative DNA helicase Dda | *Serratia* phage BF | 100 | 100 |
| Structure & packaging | pEa_SNUABM12_00313 | putative structural protein | *Serratia* phage BF | 100 | 98.89 |
| Nucleotide metabolism | pEa_SNUABM12_00315 | putative translation initiation factor IF-3 | *Serratia* phage BF | 100 | 100 |
| Structure & packaging | pEa_SNUABM12_00316 | putative ATP-dependent Clp protease | *Serratia* phage BF | 100 | 100 |
| Nucleotide metabolism | pEa_SNUABM12_00317 | putative DnaJ-like protein | *Serratia* phage BF | 100 | 100 |
| Structure & packaging | pEa_SNUABM12_00318 | putative structural protein | *Serratia* phage BF | 100 | 100 |
| Structure & packaging | pEa_SNUABM12_00319 | putative structural protein | *Serratia* phage BF | 100 | 100 |
| Structure & packaging | pEa_SNUABM12_00320 | putative membrane protein | *Serratia* phage BF | 100 | 100 |
| Nucleotide metabolism | pEa_SNUABM12_00321 | putative topoisomerase II large subunit | *Serratia* phage BF | 100 | 100 |
| Nucleotide metabolism | pEa_SNUABM12_00322 | putative DNA topoisomerase II medium subunit | *Serratia* phage BF | 100 | 100 |
| Structure & packaging | pEa_SNUABM12_00323 | putative structural protein | *Serratia* phage BF | 100 | 99.9 |
| Nucleotide metabolism | pEa_SNUABM12_00325 | putative DNA polymerase III alpha subunit | *Serratia* phage BF | 100 | 100 |
| Structure & packaging | pEa_SNUABM12_00327 | putative co-chaperonin GroES | *Serratia* phage BF | 100 | 100 |
| Nucleotide metabolism | pEa_SNUABM12_00329 | putative sliding clamp loader subunit | *Serratia* phage BF | 100 | 100 |
| Additional function | pEa_SNUABM12_00330 | putative phosphoglycolate phosphatase | *Serratia* phage BF | 100 | 100 |
| Structure & packaging | pEa_SNUABM12_00331 | putaive structural protein | *Serratia* phage BF | 100 | 100 |
| Structure & packaging | pEa_SNUABM12_00332 | putaive structural protein | *Serratia* phage BF | 100 | 100 |
| Additional function | pEa_SNUABM12_00337 | putative TelA like protein | *Serratia* phage BF | 100 | 99.74 |
| Nucleotide metabolism | pEa_SNUABM12_00338 | putative nucleotide reductase subunit C | *Serratia* phage BF | 100 | 100 |
| Additional function | pEa_SNUABM12_00339 | putative metallopeptidase | *Serratia* phage BF | 100 | 100 |
| Structure & packaging | pEa_SNUABM12_00340 | putative membrane protein | *Serratia* phage BF | 100 | 100 |
| Structure & packaging | pEa_SNUABM12_00341 | putative structural protein | *Serratia* phage BF | 100 | 100 |
| Structure & packaging | pEa_SNUABM12_00345 | putative membrane protein | *Serratia* phage BF | 100 | 100 |
| Nucleotide metabolism | pEa_SNUABM12_00359 | putative nicotinamide nucleotide adenylyltransferase | *Serratia* phage BF | 100 | 99.43 |
| Nucleotide metabolism | pEa_SNUABM12_00360 | putative nicotinamide mononucleotide transporter PnuC | *Serratia* phage BF | 100 | 100 |
| Structure & packaging | pEa_SNUABM12_00367 | putative structural protein | *Serratia* phage BF | 100 | 100 |
| tRNA | pEa_SNUABM12_00371 | tRNA-Ser |  |  |  |
| Nucleotide metabolism | pEa_SNUABM12_00376 | putative ATPase | *Serratia* phage BF | 100 | 100 |
| tRNA | pEa_SNUABM12_00377 | tRNA-Trp |  |  |  |
| Nucleotide metabolism | pEa_SNUABM12_00379 | putative nucleotidase | *Serratia* phage BF | 100 | 98.96 |
| tRNA | pEa_SNUABM12_00382 | tRNA-Thr |  |  |  |
| tRNA | pEa_SNUABM12_00384 | tRNA-Leu |  |  |  |
| tRNA related | pEa_SNUABM12_00395 | putative peptidyl-tRNA hydrolase | *Serratia* phage BF | 100 | 95.8 |
| Structure & packaging | pEa_SNUABM12_00396 | putative HNH homing endonuclease | *Yersinia* phage fHe-Yen9-04 | 100 | 96.15 |
| tRNA | pEa_SNUABM12_00397 | tRNA-Leu |  |  |  |
| Structure & packaging | pEa_SNUABM12_00398 | putative membrane protein | *Serratia* phage BF | 100 | 100 |
| tRNA | pEa_SNUABM12_00416 | tRNA-Arg |  |  |  |
| tRNA | pEa_SNUABM12_00422 | tRNA-Pyl |  |  |  |
| tRNA | pEa_SNUABM12_00424 | tRNA-Met |  |  |  |
| tRNA | pEa_SNUABM12_00430 | tRNA-Leu |  |  |  |
| tRNA | pEa_SNUABM12_00443 | tRNA-Phe |  |  |  |
| Structure & packaging | pEa_SNUABM12_00452 | putative membrane protein | *Serratia* phage BF | 100 | 100 |
| tRNA | pEa_SNUABM12_00453 | tRNA-Leu |  |  |  |
| Structure & packaging | pEa_SNUABM12_00456 | putative membrane protein | *Serratia* phage BF | 100 | 100 |
| Nucleotide metabolism | pEa_SNUABM12_00458 | putative AAA family ATPase | *Serratia* phage BF | 100 | 100 |
| tRNA | pEa_SNUABM12_00460 | tRNA-Glu |  |  |  |
| tRNA | pEa_SNUABM12_00461 | tRNA-Ser |  |  |  |
| tRNA | pEa_SNUABM12_00463 | tRNA-Ser |  |  |  |
| tRNA | pEa_SNUABM12_00464 | tRNA-Ser |  |  |  |
| Structure & packaging | pEa_SNUABM12_00466 | putative ATP-dependent Clp protease proteolytic subunit | *Serratia* phage BF | 100 | 100 |
| tRNA | pEa_SNUABM12_00469 | tRNA-Ile |  |  |  |
| tRNA | pEa_SNUABM12_00473 | tRNA-Asn |  |  |  |
| tRNA | pEa_SNUABM12_00474 | tRNA-Gln |  |  |  |
| tRNA | pEa_SNUABM12_00475 | tRNA-Gly |  |  |  |
| tRNA | pEa_SNUABM12_00480 | tRNA-Asp |  |  |  |
| Nucleotide metabolism | pEa_SNUABM12_00482 | putative DNA condensation protein | *Serratia* phage BF | 100 | 99.44 |
| Additional function | pEa_SNUABM12_00483 | putative kelch-like protein | *Serratia* phage BF | 80 | 31.56 |
| Structure & packaging | pEa_SNUABM12_00484 | putative structural protein | *Serratia* phage BF | 100 | 100 |
| Nucleotide metabolism | pEa_SNUABM12_00486 | putative DNA condensation protein | *Serratia* phage BF | 100 | 97.92 |
| Nucleotide metabolism | pEa_SNUABM12_00490 | putative DNA condensation protein | *Serratia* phage BF | 100 | 97.33 |
| Additional function | pEa_SNUABM12_00491 | putative PE-PGRS family protein | *Serratia* phage BF | 100 | 98.57 |
| Structure & packaging | pEa_SNUABM12_00494 | putative membrane protein | *Serratia* phage BF | 100 | 99.53 |
| Structure & packaging | pEa_SNUABM12_00495 | putative membrane protein | *Serratia* phage BF | 100 | 100 |
| Structure & packaging | pEa_SNUABM12_00496 | putative membrane protein | *Serratia* phage BF | 100 | 99.09 |
| Structure & packaging | pEa_SNUABM12_00497 | putative membrane protein | *Serratia* phage BF | 100 | 100 |
| Structure & packaging | pEa_SNUABM12_00498 | putative membrane protein | *Serratia* phage BF | 100 | 99.17 |
| Structure & packaging | pEa_SNUABM12_00499 | putative membrane protein | *Serratia* phage BF | 100 | 100 |
| Structure & packaging | pEa_SNUABM12_00501 | putative membrane protein | *Serratia* phage BF | 100 | 100 |
| Nucleotide metabolism | pEa_SNUABM12_00502 | putative thioredoxin | *Serratia* phage BF | 100 | 100 |
| Structure & packaging | pEa_SNUABM12_00503 | putative HNH endonuclease | *Serratia* phage BF | 100 | 99.65 |
| Structure & packaging | pEa_SNUABM12_00504 | putative structural protein | *Serratia* phage BF | 100 | 100 |
| Structure & packaging | pEa_SNUABM12_00507 | putative membrane protein | *Serratia* phage BF | 100 | 99.08 |
| Structure & packaging | pEa_SNUABM12_00509 | putative HNH endonuclease | *Serratia* phage BF | 100 | 100 |
| Additional function | pEa_SNUABM12_00517 | putative C4-type zinc finger domain-containing protein | *Serratia* phage BF | 100 | 100 |
| Structure & packaging | pEa_SNUABM12_00521 | putative membrane protein | *Serratia* phage BF | 100 | 98.61 |
| Structure & packaging | pEa_SNUABM12_00522 | putative structural protein | *Serratia* phage BF | 100 | 100 |
| Nucleotide metabolism | pEa_SNUABM12_00525 | putative RNA polymerase sigma-70 subunit | *Serratia* phage BF | 100 | 100 |
| Structure & packaging | pEa_SNUABM12_00533 | putative membrane protein | *Serratia* phage BF | 100 | 100 |
| Structure & packaging | pEa_SNUABM12_00537 | putative membrane protein | *Serratia* phage BF | 100 | 99.3 |
| Structure & packaging | pEa_SNUABM12_00538 | putative membrane protein | *Serratia* phage BF | 100 | 100 |
| Structure & packaging | pEa_SNUABM12_00544 | putative structural protein | *Serratia* phage BF | 100 | 99.2 |
| Nucleotide metabolism | pEa_SNUABM12_00545 | putative S-adenosyl-L- methionine-dependent methyltransferase | *Serratia* phage BF | 100 | 100 |
| Structure & packaging | pEa_SNUABM12_00546 | putative structural protein | *Serratia* phage BF | 100 | 99.57 |
| tRNA | pEa_SNUABM12_00547 | tRNA-Ala |  |  |  |
| tRNA | pEa_SNUABM12_00548 | tRNA-Met |  |  |  |
| tRNA | pEa_SNUABM12_00549 | tRNA-Met |  |  |  |
| tRNA | pEa_SNUABM12_00551 | tRNA-Lys |  |  |  |
| tRNA | pEa_SNUABM12_00552 | tRNA-Cys |  |  |  |
| tRNA | pEa_SNUABM12_00557 | tRNA-Tyr |  |  |  |
| Structure & packaging | pEa_SNUABM12_00561 | putative membrane protein | *Serratia* phage BF | 100 | 98.13 |
| tRNA | pEa_SNUABM12_00565 | tRNA-Lys |  |  |  |
| tRNA | pEa_SNUABM12_00567 | tRNA-Phe |  |  |  |
| tRNA | pEa_SNUABM12_00568 | tRNA-His |  |  |  |
| tRNA | pEa_SNUABM12_00573 | tRNA-Pro |  |  |  |
| tRNA | pEa_SNUABM12_00574 | tRNA-Pro |  |  |  |
| tRNA | pEa_SNUABM12_00579 | tRNA-Arg |  |  |  |
