# Supplementary material for: Characterization of Novel Erwinia amylovora Jumbo Bacteriophages from Eneladusvirus Genus
Source: Viruses. 2020 Nov 30;12(12):1373. doi: 10.3390/v12121373 (PMC7760394; doi:10.3390/v12121373)
Supplement: Supplementary file 1 [file viruses-12-01373-s001.zip › Supplementary Table S2.docx]

**Table S3.** Functional classification of ORFs in *Erwinia* phage pEa_SNUABM_47.

| **Group** | **Locus tag** | **Encoded protein** | **Related organism** | **Query cover**  **(%)** | **Identiy**  **(%)** |
| --- | --- | --- | --- | --- | --- |
| Structure & packaging | pEa_SNUABM47_00019 | putative membrane protein | *Serratia* phage BF | 100 | 100 |
| Additional function | pEa_SNUABM47_00021 | SPFH domain containing protein | *Serratia* phage BF | 100 | 100 |
| Structure & packaging | pEa_SNUABM47_00026 | putative structural protein | *Serratia* phage BF | 100 | 99.35 |
| Structure & packaging | pEa_SNUABM47_00058 | putative membrane protein | *Serratia* phage BF | 100 | 100 |
| Nucleotide metabolism | pEa_SNUABM47_00062 | putative DNA N-6-adenine-methyltransferase | *Serratia* phage BF | 100 | 100 |
| Nucleotide metabolism | pEa_SNUABM47_00063 | putative cytitidyltransferase | *Serratia* phage BF | 97 | 99.42 |
| Nucleotide metabolism | pEa_SNUABM47_00071 | putative RNA ligase, T4 RnlA family | *Serratia* phage BF | 100 | 99.48 |
| Nucleotide metabolism | pEa_SNUABM47_00075 | putative polynucleotide 5'-kinase and 3'-phosphatase | *Serratia* phage BF | 100 | 96.75 |
| Structure & packaging | pEa_SNUABM47_00079 | putative membrane protein | *Serratia* phage BF | 100 | 100 |
| Structure & packaging | pEa_SNUABM47_00082 | putative membrane protein | *Serratia* phage BF | 100 | 98.55 |
| Additional function | pEa_SNUABM47_00089 | putative serine/threonine-protein phosphatase | *Serratia* phage BF | 100 | 100 |
| Nucleotide metabolism | pEa_SNUABM47_00092 | putative DNA ligase | *Serratia* phage BF | 100 | 99.79 |
| Additional function | pEa_SNUABM47_00094 | putative serine/threonine-protein phosphatase | *Serratia* phage BF | 100 | 99.25 |
| Structure & packaging | pEa_SNUABM47_00096 | putative ATP-dependent Clp protease proteolytic subunit | *Serratia* phage BF | 100 | 100 |
| Structure & packaging | pEa_SNUABM47_00101 | putative structural protein | *Serratia* phage BF | 100 | 100 |
| Nucleotide metabolism | pEa_SNUABM47_00102 | putative CMP/dCMP deaminase | *Serratia* phage BF | 100 | 98.72 |
| Structure & packaging | pEa_SNUABM47_00104 | putative major tail protein | *Serratia* phage BF | 100 | 98.34 |
| Structure & packaging | pEa_SNUABM47_00105 | putative major tail protein | *Serratia* phage BF | 100 | 100 |
| Nucleotide metabolism | pEa_SNUABM47_00107 | putative nicotinamide phosphoribosyltransferase | *Serratia* phage BF | 100 | 99.8 |
| Structure & packaging | pEa_SNUABM47_00108 | putative membrane protein | *Serratia* phage BF | 100 | 100 |
| Structure & packaging | pEa_SNUABM47_00109 | putative structural protein | *Serratia* phage BF | 100 | 98.65 |
| Nucleotide metabolism | pEa_SNUABM47_00111 | putative Sir2-like protein | *Serratia* phage BF | 100 | 100 |
| Nucleotide metabolism | pEa_SNUABM47_00112 | putative nudix hydrolase | *Serratia* phage BF | 100 | 100 |
| Structure & packaging | pEa_SNUABM47_00113 | putative membrane protein | *Serratia* phage BF | 100 | 100 |
| Additional function | pEa_SNUABM47_00114 | putative PhoH family protein | *Serratia* phage BF | 100 | 100 |
| Structure & packaging | pEa_SNUABM47_00115 | putative prohead core scaffolding protein | *Serratia* phage BF | 100 | 98.7 |
| Structure & packaging | pEa_SNUABM47_00118 | putative membrane protein | *Serratia* phage BF | 98 | 100 |
| Structure & packaging | pEa_SNUABM47_00119 | putative membrane protein | *Serratia* phage BF | 100 | 100 |
| Structure & packaging | pEa_SNUABM47_00123 | putative co-chaperonin GroES | *Serratia* phage BF | 100 | 100 |
| tRNA related | pEa_SNUABM47_00124 | putative tyrosyl-tRNA synthetase | *Serratia* phage BF | 100 | 99.76 |
| Nucleotide metabolism | pEa_SNUABM47_00128 | putative adenine-specific DNA methylase | *Serratia* phage BF | 100 | 100 |
| Nucleotide metabolism | pEa_SNUABM47_00130 | putative dCMP deaminase | *Serratia* phage BF | 100 | 100 |
| Nucleotide metabolism | pEa_SNUABM47_00131 | putative AAA domain-containing ATPase | *Serratia* phage BF | 100 | 99.46 |
| Nucleotide metabolism | pEa_SNUABM47_00132 | putative anaerobic NTP reductase large subunit | *Serratia* phage BF | 100 | 100 |
| Nucleotide metabolism | pEa_SNUABM47_00136 | putative pyruvate formate-lyase | *Serratia* phage BF | 100 | 100 |
| Nucleotide metabolism | pEa_SNUABM47_00138 | putative anaerobic ribonucleoside-triphosphate reductase activating protein | *Serratia* phage BF | 100 | 99.36 |
| Structure & packaging | pEa_SNUABM47_00140 | putative minor tail protein | *Serratia* phage BF | 100 | 98.00 |
| Structure & packaging | pEa_SNUABM47_00141 | putative structural protein | *Serratia* phage BF | 100 | 99.89 |
| Structure & packaging | pEa_SNUABM47_00142 | putative structural protein | *Serratia* phage BF | 100 | 99.35 |
| tRNA related | pEa_SNUABM47_00145 | putative tRNAHis-5'-guanylyltransferase | *Serratia* phage BF | 100 | 99.61 |
| Nucleotide metabolism | pEa_SNUABM47_00147 | putative NrdA protein | *Serratia* phage BF | 98 | 98.99 |
| Nucleotide metabolism | pEa_SNUABM47_00150 | putative site specific DNA methyltransferase | *Serratia* phage BF | 100 | 99.3 |
| Nucleotide metabolism | pEa_SNUABM47_00152 | putative nucleotidyltransferase | *Serratia* phage BF | 100 | 100 |
| Additional function | pEa_SNUABM47_00157 | putative acyl carrier protein | *Serratia* phage BF | 100 | 100 |
| Structure & packaging | pEa_SNUABM47_00158 | putative membrane protein | *Serratia* phage BF | 100 | 99.17 |
| Lysis | pEa_SNUABM47_00159 | putative o-spannin | *Serratia* phage BF | 100 | 99.03 |
| Structure & packaging | pEa_SNUABM47_00160 | putative structural protein | *Serratia* phage BF | 100 | 100 |
| Nucleotide metabolism | pEa_SNUABM47_00163 | putative starvation-inducible DNA-binding protein | *Serratia* phage BF | 100 | 100 |
| Nucleotide metabolism | pEa_SNUABM47_00167 | putative GTP cyclohydrolase | *Serratia* phage BF | 100 | 100 |
| Nucleotide metabolism | pEa_SNUABM47_00169 | putative thymidine kinase | *Serratia* phage BF | 100 | 99.48 |
| Structure & packaging | pEa_SNUABM47_00170 | putative tail protein | *Serratia* phage BF | 100 | 100 |
| Structure & packaging | pEa_SNUABM47_00171 | putative membrane protein | *Serratia* phage BF | 100 | 100 |
| Structure & packaging | pEa_SNUABM47_00173 | putative membrane protein | *Yersinia* phage fHe-Yen9-03 | 100 | 72.73 |
| Nucleotide metabolism | pEa_SNUABM47_00175 | putative RNA ligase | *Serratia* phage BF | 100 | 99.52 |
| Structure & packaging | pEa_SNUABM47_00179 | putative membrane protein | *Serratia* phage BF | 100 | 97.83 |
| Lysis | pEa_SNUABM47_00182 | putative lysozyme | *Serratia* phage BF | 100 | 100 |
| Nucleotide metabolism | pEa_SNUABM47_00183 | putative sigma 54 modulation protein/ribosomal protein | *Serratia* phage BF | 100 | 100 |
| tRNA related | pEa_SNUABM47_00185 | putative tRNA nucleotidyl transferase | *Serratia* phage BF | 100 | 100 |
| Nucleotide metabolism | pEa_SNUABM47_00186 | putative nudix hydrolase | *Serratia* phage BF | 100 | 100 |
| Nucleotide metabolism | pEa_SNUABM47_00187 | putative glutaredoxin | *Serratia* phage BF | 100 | 100 |
| tRNA related | pEa_SNUABM47_00188 | putative aspartyl-tRNA amidotransferase | *Serratia* phage BF | 100 | 100 |
| Nucleotide metabolism | pEa_SNUABM47_00191 | putative Appr-1-p processing enzyme | *Serratia* phage BF | 100 | 99.4 |
| Structure & packaging | pEa_SNUABM47_00194 | putative neck protein | *Serratia* phage BF | 100 | 100 |
| Nucleotide metabolism | pEa_SNUABM47_00196 | putative deoxynucleotide monophosphate kinase | *Serratia* phage BF | 100 | 99.12 |
| Structure & packaging | pEa_SNUABM47_00197 | putative tail sheath protein | *Serratia* phage BF | 100 | 99.89 |
| Structure & packaging | pEa_SNUABM47_00198 | putative structural protein | *Yersinia* phage fHe-Yen9-04 | 100 | 93.45 |
| Structure & packaging | pEa_SNUABM47_00199 | putative structural protein | *Serratia* phage BF | 100 | 100 |
| Structure & packaging | pEa_SNUABM47_00200 | putative structural protein | *Serratia* phage BF | 100 | 100 |
| Structure & packaging | pEa_SNUABM47_00201 | putative head completion protein | *Serratia* phage BF | 100 | 100 |
| Structure & packaging | pEa_SNUABM47_00203 | putative structural protein | *Serratia* phage BF | 100 | 99.45 |
| Structure & packaging | pEa_SNUABM47_00204 | putative structural protein | *Serratia* phage BF | 100 | 100 |
| Structure & packaging | pEa_SNUABM47_00205 | putative structural protein | *Serratia* phage BF | 100 | 100 |
| Structure & packaging | pEa_SNUABM47_00206 | putative structural protein | *Serratia* phage BF | 100 | 99.38 |
| Structure & packaging | pEa_SNUABM47_00207 | putative structural protein | *Serratia* phage BF | 100 | 100 |
| Nucleotide metabolism | pEa_SNUABM47_00209 | putative ATPase | *Serratia* phage BF | 100 | 100 |
| Nucleotide metabolism | pEa_SNUABM47_00216 | putative thymidylate synthase | *Serratia* phage BF | 100 | 100 |
| Structure & packaging | pEa_SNUABM47_00217 | putative structural protein | *Serratia* phage BF | 100 | 100 |
| Structure & packaging | pEa_SNUABM47_00221 | putative structural protein | *Serratia* phage BF | 100 | 100 |
| Structure & packaging | pEa_SNUABM47_00222 | putative long tail fiber proximal subunit | *Serratia* phage BF | 100 | 97.52 |
| Structure & packaging | pEa_SNUABM47_00223 | putative structural protein | *Serratia* phage BF | 100 | 100 |
| Structure & packaging | pEa_SNUABM47_00224 | putative structural protein | *Serratia* phage BF | 100 | 100 |
| Structure & packaging | pEa_SNUABM47_00225 | putative structural protein | *Serratia* phage BF | 100 | 100 |
| Structure & packaging | pEa_SNUABM47_00226 | putative structural protein | *Serratia* phage BF | 100 | 100 |
| Structure & packaging | pEa_SNUABM47_00227 | putative structural protein | *Serratia* phage BF | 100 | 100 |
| Nucleotide metabolism | pEa_SNUABM47_00228 | putative NUDIX hydrolase family protein | *Serratia* phage BF | 100 | 100 |
| Structure & packaging | pEa_SNUABM47_00229 | putative tail sheath stabilizer and completion protein | *Serratia* phage BF | 100 | 100 |
| Structure & packaging | pEa_SNUABM47_00230 | putative structural protein | *Serratia* phage BF | 100 | 100 |
| Structure & packaging | pEa_SNUABM47_00231 | putative ATP-dependent Clp protease ATP-binding subunit clpA | *Serratia* phage BF | 100 | 99.74 |
| Structure & packaging | pEa_SNUABM47_00232 | putaive structural protein | *Serratia* phage BF | 100 | 99.88 |
| Structure & packaging | pEa_SNUABM47_00233 | putative baseplate wedge | *Serratia* phage BF | 100 | 99.83 |
| Structure & packaging | pEa_SNUABM47_00234 | putative baseplate protein | *Serratia* phage BF | 100 | 100 |
| Lysis | pEa_SNUABM47_00235 | putative baseplate hub subunit and tail lysozyme | *Serratia* phage BF | 100 | 100 |
| Lysis | pEa_SNUABM47_00236 | putative T4-like phage baseplate hub and tail lysozyme | *Serratia* phage BF | 100 | 100 |
| Structure & packaging | pEa_SNUABM47_00237 | putative structural protein | *Serratia* phage BF | 100 | 100 |
| Structure & packaging | pEa_SNUABM47_00238 | putative baseplate wedge protein | *Serratia* phage BF | 100 | 99.04 |
| Structure & packaging | pEa_SNUABM47_00239 | putative structural protein | *Serratia* phage BF | 100 | 100 |
| Nucleotide metabolism | pEa_SNUABM47_00240 | putative RNA sigma factor for late transcription | *Serratia* phage BF | 100 | 100 |
| Nucleotide metabolism | pEa_SNUABM47_00241 | putative endonuclease subunit | *Serratia* phage BF | 100 | 100 |
| Nucleotide metabolism | pEa_SNUABM47_00242 | putative endonuclease subunit | *Serratia* phage BF | 100 | 99.72 |
| Structure & packaging | pEa_SNUABM47_00243 | putative EndoVII packaging and recombination endonuclease | *Serratia* phage BF | 100 | 100 |
| Structure & packaging | pEa_SNUABM47_00245 | putative baseplate hub subunit | *Serratia* phage BF | 100 | 100 |
| Structure & packaging | pEa_SNUABM47_00246 | putative tape measure protein | *Serratia* phage BF | 100 | 99.86 |
| Structure & packaging | pEa_SNUABM47_00247 | putative portal vertex protein | *Serratia* phage BF | 100 | 100 |
| Structure & packaging | pEa_SNUABM47_00249 | putative structural protein | *Serratia* phage BF | 100 | 99.66 |
| Structure & packaging | pEa_SNUABM47_00250 | putative prohead core protein | *Serratia* phage BF | 100 | 100 |
| Structure & packaging | pEa_SNUABM47_00251 | putative scaffolding protein | *Serratia* phage BF | 100 | 100 |
| Structure & packaging | pEa_SNUABM47_00252 | putative major capsid protein | *Serratia* phage BF | 100 | 100 |
| Nucleotide metabolism | pEa_SNUABM47_00254 | putative GIY-YIG nuclease family protein | *Serratia* phage BF | 100 | 100 |
| Structure & packaging | pEa_SNUABM47_00255 | putative tail fiber protein | *Serratia* phage BF | 100 | 99.82 |
| Nucleotide metabolism | pEa_SNUABM47_00258 | putative DNA polymerase | *Serratia* phage BF | 100 | 99.9 |
| Additional function | pEa_SNUABM47_00261 | putative serine/threonine protein phosphatase | *Serratia* phage BF | 100 | 100 |
| Additional function | pEa_SNUABM47_00262 | putative type I antifreeze protein | *Yersinia* phage fHe-Yen9-04 | 100 | 85.51 |
| Structure & packaging | pEa_SNUABM47_00263 | putative co-chaperonin GroES | *Serratia* phage BF | 100 | 100 |
| Structure & packaging | pEa_SNUABM47_00264 | putative structural protein | *Serratia* phage BF | 100 | 100 |
| Structure & packaging | pEa_SNUABM47_00265 | putative structural protein | *Serratia* phage BF | 100 | 100 |
| Structure & packaging | pEa_SNUABM47_00266 | putative structural protein | *Serratia* phage BF | 100 | 100 |
| Nucleotide metabolism | pEa_SNUABM47_00267 | putative RNaseH ribonuclease | *Serratia* phage BF | 100 | 100 |
| Structure & packaging | pEa_SNUABM47_00269 | putative terminase like protein | *Serratia* phage BF | 100 | 99.58 |
| Structure & packaging | pEa_SNUABM47_00270 | putative terminase large subunit | *Serratia* phage BF | 100 | 100 |
| Structure & packaging | pEa_SNUABM47_00271 | putative structural protein | *Serratia* phage BF | 100 | 99.77 |
| Nucleotide metabolism | pEa_SNUABM47_00272 | putative ssDNA binding protein | *Serratia* phage BF | 100 | 100 |
| Nucleotide metabolism | pEa_SNUABM47_00273 | putative UvsX protein | *Serratia* phage BF | 100 | 100 |
| Nucleotide metabolism | pEa_SNUABM47_00274 | putative UvsY portein | *Serratia* phage BF | 100 | 100 |
| Nucleotide metabolism | pEa_SNUABM47_00275 | putative DNA polymerase III epsilon subunit | *Serratia* phage BF | 100 | 100 |
| Nucleotide metabolism | pEa_SNUABM47_00276 | putative RNA-DNA + DNA-DNA helicase | *Serratia* phage BF | 100 | 100 |
| Structure & packaging | pEa_SNUABM47_00281 | putative structural protein | *Serratia* phage BF | 100 | 100 |
| Structure & packaging | pEa_SNUABM47_00282 | putative membrane protein | *Serratia* phage BF | 100 | 100 |
| Structure & packaging | pEa_SNUABM47_00284 | putative structural protein | *Serratia* phage BF | 100 | 100 |
| Nucleotide metabolism | pEa_SNUABM47_00285 | putative DNA primase subunit | *Serratia* phage BF | 100 | 99.71 |
| Nucleotide metabolism | pEa_SNUABM47_00286 | putative DNA primase-helicase | *Serratia* phage BF | 100 | 100 |
| Structure & packaging | pEa_SNUABM47_00290 | putative structural protein | *Serratia* phage BF | 100 | 99.67 |
| Structure & packaging | pEa_SNUABM47_00291 | putative structural protein | *Serratia* phage BF | 100 | 100 |
| Structure & packaging | pEa_SNUABM47_00293 | putative structural protein | *Serratia* phage BF | 100 | 99.54 |
| Structure & packaging | pEa_SNUABM47_00294 | putative structural protein | *Serratia* phage BF | 100 | 100 |
| Nucleotide metabolism | pEa_SNUABM47_00295 | putative restriction endonuclease type II like-protein | *Serratia* phage BF | 100 | 100 |
| Structure & packaging | pEa_SNUABM47_00297 | putative structural protein | *Serratia* phage BF | 100 | 100 |
| Nucleotide metabolism | pEa_SNUABM47_00298 | putative aerobic ribonucleotide-diphosphate reductase alpha subunit | *Serratia* phage BF | 100 | 100 |
| Nucleotide metabolism | pEa_SNUABM47_00299 | putative aerobic ribonucleotide-diphosphate reductase beta subunit | *Serratia* phage BF | 100 | 100 |
| Structure & packaging | pEa_SNUABM47_00300 | putative membrane protein | *Serratia* phage BF | 100 | 100 |
| Structure & packaging | pEa_SNUABM47_00302 | putative structural protein | *Serratia* phage BF | 100 | 100 |
| Nucleotide metabolism | pEa_SNUABM47_00303 | putative nucleotide pyrophosphohydrolase | *Serratia* phage BF | 100 | 99.57 |
| Structure & packaging | pEa_SNUABM47_00305 | putative structural protein | *Serratia* phage BF | 100 | 100 |
| Structure & packaging | pEa_SNUABM47_00306 | putative membrane protein | *Serratia* phage BF | 100 | 100 |
| Nucleotide metabolism | pEa_SNUABM47_00307 | putative dihydrofolate reductase | *Serratia* phage BF | 100 | 98.28 |
| Nucleotide metabolism | pEa_SNUABM47_00308 | putative ribonuclease H | *Serratia* phage BF | 100 | 100 |
| Nucleotide metabolism | pEa_SNUABM47_00309 | putative DNA helicase Dda | *Serratia* phage BF | 100 | 100 |
| Structure & packaging | pEa_SNUABM47_00311 | putative structural protein | *Serratia* phage BF | 100 | 98.89 |
| Nucleotide metabolism | pEa_SNUABM47_00313 | putative translation initiation factor IF-3 | *Serratia* phage BF | 100 | 100 |
| Structure & packaging | pEa_SNUABM47_00314 | putative ATP-dependent Clp protease | *Serratia* phage BF | 100 | 100 |
| Nucleotide metabolism | pEa_SNUABM47_00315 | putative DnaJ-like protein | *Serratia* phage BF | 100 | 100 |
| Structure & packaging | pEa_SNUABM47_00316 | putative structural protein | *Serratia* phage BF | 100 | 99.81 |
| Structure & packaging | pEa_SNUABM47_00317 | putative structural protein | *Serratia* phage BF | 100 | 99.75 |
| Structure & packaging | pEa_SNUABM47_00318 | putative membrane protein | *Serratia* phage BF | 100 | 100 |
| Nucleotide metabolism | pEa_SNUABM47_00319 | putative topoisomerase II large subunit | *Serratia* phage BF | 100 | 100 |
| Nucleotide metabolism | pEa_SNUABM47_00320 | putative DNA topoisomerase II medium subunit | *Serratia* phage BF | 100 | 100 |
| Structure & packaging | pEa_SNUABM47_00321 | putative structural protein | *Serratia* phage BF | 100 | 99.9 |
| Nucleotide metabolism | pEa_SNUABM47_00323 | putative DNA polymerase III alpha subunit | *Serratia* phage BF | 100 | 100 |
| Structure & packaging | pEa_SNUABM47_00325 | putative co-chaperonin GroES | *Serratia* phage BF | 100 | 100 |
| Nucleotide metabolism | pEa_SNUABM47_00327 | putative sliding clamp loader subunit | *Serratia* phage BF | 100 | 100 |
| Additional function | pEa_SNUABM47_00328 | putative phosphoglycolate phosphatase | *Serratia* phage BF | 100 | 100 |
| Structure & packaging | pEa_SNUABM47_00329 | putaive structural protein | *Serratia* phage BF | 100 | 100 |
| Structure & packaging | pEa_SNUABM47_00330 | putaive structural protein | *Serratia* phage BF | 100 | 100 |
| Additional function | pEa_SNUABM47_00335 | putative TelA like protein | *Serratia* phage BF | 100 | 99.74 |
| Nucleotide metabolism | pEa_SNUABM47_00336 | putative nucleotide reductase subunit C | *Serratia* phage BF | 100 | 100 |
| Additional function | pEa_SNUABM47_00337 | putative metallopeptidase | *Serratia* phage BF | 100 | 100 |
| Structure & packaging | pEa_SNUABM47_00338 | putative membrane protein | *Serratia* phage BF | 100 | 100 |
| Structure & packaging | pEa_SNUABM47_00339 | putative structural protein | *Serratia* phage BF | 100 | 100 |
| Structure & packaging | pEa_SNUABM47_00343 | putative membrane protein | *Serratia* phage BF | 100 | 100 |
| Nucleotide metabolism | pEa_SNUABM47_00346 | putative ADP-ribosylglycohydrolase | *Serratia* phage BF | 100 | 98.71 |
| Nucleotide metabolism | pEa_SNUABM47_00356 | putative nicotinamide nucleotide adenylyltransferase | *Serratia* phage BF | 100 | 99.72 |
| Nucleotide metabolism | pEa_SNUABM47_00357 | putative nicotinamide mononucleotide transporter PnuC | *Serratia* phage BF | 100 | 100 |
| Structure & packaging | pEa_SNUABM47_00364 | putative structural protein | *Serratia* phage BF | 100 | 100 |
| tRNA | pEa_SNUABM47_00368 | tRNA-Ser |  |  |  |
| Nucleotide metabolism | pEa_SNUABM47_00373 | putative ATPase | *Serratia* phage BF | 100 | 100 |
| tRNA | pEa_SNUABM47_00374 | tRNA-Trp |  |  |  |
| Nucleotide metabolism | pEa_SNUABM47_00376 | putative nucleotidase | *Serratia* phage BF | 100 | 100 |
| tRNA | pEa_SNUABM47_00379 | tRNA-Thr |  |  |  |
| tRNA | pEa_SNUABM47_00381 | tRNA-Leu |  |  |  |
| tRNA related | pEa_SNUABM47_00392 | putative peptidyl-tRNA hydrolase | *Serratia* phage BF | 100 | 100 |
| tRNA | pEa_SNUABM47_00393 | tRNA-Leu |  |  |  |
| Structure & packaging | pEa_SNUABM47_00394 | putative membrane protein | *Serratia* phage BF | 100 | 100 |
| tRNA | pEa_SNUABM47_00412 | tRNA-Arg |  |  |  |
| tRNA | pEa_SNUABM47_00417 | tRNA-Pyl |  |  |  |
| tRNA | pEa_SNUABM47_00420 | tRNA-Met |  |  |  |
| tRNA | pEa_SNUABM47_00426 | tRNA-Leu |  |  |  |
| tRNA | pEa_SNUABM47_00439 | tRNA-Phe |  |  |  |
| tRNA | pEa_SNUABM47_00444 | tRNA-Lys |  |  |  |
| Structure & packaging | pEa_SNUABM47_00448 | putative membrane protein | *Serratia* phage BF | 100 | 99.12 |
| tRNA | pEa_SNUABM47_00449 | tRNA-Leu |  |  |  |
| Structure & packaging | pEa_SNUABM47_00452 | putative membrane protein | *Serratia* phage BF | 100 | 100 |
| Nucleotide metabolism | pEa_SNUABM47_00454 | putative AAA family ATPase | *Serratia* phage BF | 100 | 100 |
| tRNA | pEa_SNUABM47_00456 | tRNA-Glu |  |  |  |
| tRNA | pEa_SNUABM47_00457 | tRNA-Ser |  |  |  |
| tRNA | pEa_SNUABM47_00459 | tRNA-Ser |  |  |  |
| tRNA | pEa_SNUABM47_00460 | tRNA-Ser |  |  |  |
| Structure & packaging | pEa_SNUABM47_00462 | putative ATP-dependent Clp protease proteolytic subunit | *Serratia* phage BF | 100 | 100 |
| tRNA | pEa_SNUABM47_00465 | tRNA-Ile |  |  |  |
| tRNA | pEa_SNUABM47_00469 | tRNA-Asn |  |  |  |
| tRNA | pEa_SNUABM47_00470 | tRNA-Gln |  |  |  |
| tRNA | pEa_SNUABM47_00471 | tRNA-Gly |  |  |  |
| tRNA | pEa_SNUABM47_00476 | tRNA-Asp |  |  |  |
| tRNA | pEa_SNUABM47_00478 | tRNA-Arg |  |  |  |
| tRNA | pEa_SNUABM47_00483 | tRNA-Pro |  |  |  |
| tRNA | pEa_SNUABM47_00484 | tRNA-Pro |  |  |  |
| tRNA | pEa_SNUABM47_00485 | tRNA-Pro |  |  |  |
| tRNA | pEa_SNUABM47_00486 | tRNA-Val |  |  |  |
| tRNA | pEa_SNUABM47_00489 | tRNA-His |  |  |  |
| tRNA | pEa_SNUABM47_00490 | tRNA-Phe |  |  |  |
| tRNA | pEa_SNUABM47_00492 | tRNA-Lys |  |  |  |
| Structure & packaging | pEa_SNUABM47_00496 | putative membrane protein | *Serratia* phage BF | 100 | 98.13 |
| Structure & packaging | pEa_SNUABM47_00497 | putative membrane protein | *Serratia* phage BF | 100 | 99.03 |
| tRNA | pEa_SNUABM47_00500 | tRNA-Tyr |  |  |  |
| tRNA | pEa_SNUABM47_00505 | tRNA-Cys |  |  |  |
| tRNA | pEa_SNUABM47_00506 | tRNA-Lys |  |  |  |
| tRNA | pEa_SNUABM47_00508 | tRNA-Met |  |  |  |
| tRNA | pEa_SNUABM47_00509 | tRNA-Met |  |  |  |
| tRNA | pEa_SNUABM47_00510 | tRNA-Ala |  |  |  |
| Structure & packaging | pEa_SNUABM47_00511 | putative structural protein | *Serratia* phage BF | 100 | 99.57 |
| Nucleotide metabolism | pEa_SNUABM47_00512 | putative S-adenosyl-L- methionine-dependent methyltransferase | *Serratia* phage BF | 100 | 100 |
| Structure & packaging | pEa_SNUABM47_00513 | putative structural protein | *Serratia* phage BF | 100 | 99.47 |
| Structure & packaging | pEa_SNUABM47_00519 | putative membrane protein | *Serratia* phage BF | 100 | 100 |
| Structure & packaging | pEa_SNUABM47_00520 | putative membrane protein | *Serratia* phage BF | 100 | 99.3 |
| Nucleotide metabolism | pEa_SNUABM47_00532 | putative subfamily RNA polymerase sigma-70 subunit | *Serratia* phage BF | 100 | 100 |
| Structure & packaging | pEa_SNUABM47_00535 | putative structural protein | *Serratia* phage BF | 100 | 100 |
| Structure & packaging | pEa_SNUABM47_00536 | putative membrane protein | *Serratia* phage BF | 100 | 98.61 |
| Additional function | pEa_SNUABM47_00540 | putative C4-type zinc finger domain-containing protein | *Serratia* phage BF | 100 | 98.84 |
| Structure & packaging | pEa_SNUABM47_00548 | putative HNH endonuclease | *Serratia* phage BF | 100 | 100 |
| Structure & packaging | pEa_SNUABM47_00554 | putative HNH endonuclease | *Serratia* phage BF | 100 | 99.65 |
| Nucleotide metabolism | pEa_SNUABM47_00555 | putative thioredoxin | *Serratia* phage BF | 100 | 100 |
| Structure & packaging | pEa_SNUABM47_00556 | putative membrane protein | *Serratia* phage BF | 100 | 100 |
| Structure & packaging | pEa_SNUABM47_00558 | putative membrane protein | *Serratia* phage BF | 100 | 100 |
| Structure & packaging | pEa_SNUABM47_00559 | putative membrane protein | *Serratia* phage BF | 100 | 97.5 |
| Structure & packaging | pEa_SNUABM47_00560 | putative membrane protein | *Serratia* phage BF | 100 | 100 |
| Structure & packaging | pEa_SNUABM47_00561 | putative membrane protein | *Serratia* phage BF | 100 | 98.18 |
| Structure & packaging | pEa_SNUABM47_00562 | putative membrane protein | *Serratia* phage BF | 100 | 100 |
| Structure & packaging | pEa_SNUABM47_00563 | putative membrane protein | *Serratia* phage BF | 100 | 100 |
| Additional function | pEa_SNUABM47_00566 | putative PE-PGRS family protein | *Serratia* phage BF | 100 | 99.29 |
| Nucleotide metabolism | pEa_SNUABM47_00567 | putative DNA condensation protein | *Serratia* phage BF | 100 | 97.86 |
| Nucleotide metabolism | pEa_SNUABM47_00571 | putative DNA condensation protein | *Serratia* phage BF | 100 | 97.66 |
| Nucleotide metabolism | pEa_SNUABM47_00572 | putative DNA condensation protein | *Serratia* phage BF | 100 | 98.44 |
| Structure & packaging | pEa_SNUABM47_00574 | putative structural protein | *Serratia* phage BF | 100 | 100 |
| Nucleotide metabolism | pEa_SNUABM47_00575 | putative DNA condensation protein | *Serratia* phage BF | 100 | 99.44 |
