# Supplementary material for: Characterization of Novel Erwinia amylovora Jumbo Bacteriophages from Eneladusvirus Genus
Source: Viruses. 2020 Nov 30;12(12):1373. doi: 10.3390/v12121373 (PMC7760394; doi:10.3390/v12121373)
Supplement: Supplementary file 1 [file viruses-12-01373-s001.zip › Supplementary Table S3.docx]

**Table S4.** Functional classification of ORFs in *Erwinia* phage pEa_SNUABM_50.

| **Group** | **Locus tag** | **Encoded protein** | **Related organism** | **Query cover**  **(%)** | **Identiy**  **(%)** |
| --- | --- | --- | --- | --- | --- |
| Structure & packaging | pEa_SNUABM50_00019 | putative membrane protein | *Serratia* phage BF | 100 | 100 |
| Additional function | pEa_SNUABM50_00021 | SPFH domain containing protein | *Serratia* phage BF | 100 | 100 |
| Structure & packaging | pEa_SNUABM50_00025 | putative structural protein | *Serratia* phage BF | 100 | 98.71 |
| Structure & packaging | pEa_SNUABM50_00061 | putative DNA N-6-adenine-methyltransferase | *Serratia* phage BF | 100 | 100 |
| Nucleotide metabolism | pEa_SNUABM50_00062 | putative cytitidyltransferase | *Serratia* phage BF | 97 | 100 |
| Nucleotide metabolism | pEa_SNUABM50_00070 | putative RNA ligase, T4 RnlA family | *Serratia* phage BF | 100 | 99.74 |
| Nucleotide metabolism | pEa_SNUABM50_00074 | putative polynucleotide 5'-kinase and 3'-phosphatase | *Serratia* phage BF | 100 | 96.75 |
| Structure & packaging | pEa_SNUABM50_00078 | putative membrane protein | *Serratia* phage BF | 100 | 100 |
| Structure & packaging | pEa_SNUABM50_00081 | putative membrane protein | *Serratia* phage BF | 100 | 100 |
| Additional function | pEa_SNUABM50_00088 | putative serine/threonine-protein phosphatase | *Serratia* phage BF | 100 | 100 |
| Nucleotide metabolism | pEa_SNUABM50_00091 | putative DNA ligase | *Serratia* phage BF | 100 | 99.79 |
| Additional function | pEa_SNUABM50_00093 | putative serine/threonine-protein phosphatase | *Serratia* phage BF | 100 | 99.25 |
| Structure & packaging | pEa_SNUABM50_00095 | putative ATP-dependent Clp protease proteolytic subunit | *Serratia* phage BF | 100 | 100 |
| Structure & packaging | pEa_SNUABM50_00100 | putative structural protein | *Serratia* phage BF | 100 | 100 |
| Nucleotide metabolism | pEa_SNUABM50_00101 | putative CMP/dCMP deaminase | *Serratia* phage BF | 100 | 99.36 |
| Structure & packaging | pEa_SNUABM50_00103 | putative major tail protein | *Serratia* phage BF | 100 | 99.34 |
| Nucleotide metabolism | pEa_SNUABM50_00104 | putative bifunctional nicotinamide mononucleotide adenylyltransferase/ADP-ribose pyrophosphatase | *Serratia* phage BF | 100 | 100 |
| Nucleotide metabolism | pEa_SNUABM50_00106 | putative nicotinamide phosphoribosyltransferase | *Serratia* phage BF | 100 | 99.8 |
| Structure & packaging | pEa_SNUABM50_00107 | putative membrane protein | *Serratia* phage BF | 100 | 100 |
| Structure & packaging | pEa_SNUABM50_00108 | putative structural protein | *Serratia* phage BF | 100 | 98.65 |
| Nucleotide metabolism | pEa_SNUABM50_00110 | putative Sir2-like protein | *Serratia* phage BF | 100 | 100 |
| Nucleotide metabolism | pEa_SNUABM50_00111 | putative nudix hydrolase | *Serratia* phage BF | 100 | 100 |
| Structure & packaging | pEa_SNUABM50_00112 | putative membrane protein | *Serratia* phage BF | 100 | 100 |
| Additional function | pEa_SNUABM50_00113 | putative PhoH family protein | *Serratia* phage BF | 100 | 100 |
| Structure & packaging | pEa_SNUABM50_00114 | putative prohead core scaffolding protein | *Serratia* phage BF | 100 | 99.35 |
| Structure & packaging | pEa_SNUABM50_00117 | putative membrane protein | *Serratia* phage BF | 100 | 100 |
| Structure & packaging | pEa_SNUABM50_00118 | putative membrane protein | *Serratia* phage BF | 100 | 100 |
| Structure & packaging | pEa_SNUABM50_00122 | putative co-chaperonin GroES | *Serratia* phage BF | 100 | 98.91 |
| tRNA related | pEa_SNUABM50_00123 | putative tyrosyl-tRNA synthetase | *Serratia* phage BF | 100 | 99.76 |
| Nucleotide metabolism | pEa_SNUABM50_00127 | putative adenine-specific DNA methylase | *Serratia* phage BF | 100 | 100 |
| Nucleotide metabolism | pEa_SNUABM50_00129 | putative dCMP deaminase | *Serratia* phage BF | 100 | 100 |
| Nucleotide metabolism | pEa_SNUABM50_00130 | putative AAA domain-containing ATPase | *Serratia* phage BF | 100 | 100 |
| Nucleotide metabolism | pEa_SNUABM50_00131 | putative anaerobic NTP reductase large subunit | *Serratia* phage BF | 100 | 100 |
| Nucleotide metabolism | pEa_SNUABM50_00135 | putative Pyruvate formate-lyase | *Serratia* phage BF | 100 | 100 |
| Nucleotide metabolism | pEa_SNUABM50_00137 | putative anaerobic ribonucleoside-triphosphate reductase activating protein | *Serratia* phage BF | 100 | 98.73 |
| Structure & packaging | pEa_SNUABM50_00139 | putative minor tail protein | *Serratia* phage BF | 100 | 98.89 |
| Structure & packaging | pEa_SNUABM50_00140 | putative structural protein | *Serratia* phage BF | 100 | 99.89 |
| Structure & packaging | pEa_SNUABM50_00141 | putative structural protein | *Serratia* phage BF | 100 | 99.57 |
| tRNA related | pEa_SNUABM50_00144 | putative tRNAHis-5'-guanylyltransferase | *Serratia* phage BF | 100 | 99.61 |
| Nucleotide metabolism | pEa_SNUABM50_00146 | putative NrdA protein | *Serratia* phage BF | 98 | 98.99 |
| Nucleotide metabolism | pEa_SNUABM50_00149 | putative site specific DNA methyltransferase | *Serratia* phage BF | 100 | 99.3 |
| Nucleotide metabolism | pEa_SNUABM50_00151 | putative nucleotidyltransferase | *Serratia* phage BF | 100 | 100 |
| Additional function | pEa_SNUABM50_00156 | putative acyl carrier protein | *Serratia* phage BF | 100 | 100 |
| Structure & packaging | pEa_SNUABM50_00157 | putative membrane protein | *Serratia* phage BF | 100 | 100 |
| Lysis | pEa_SNUABM50_00158 | putative o-spannin | *Serratia* phage BF | 100 | 99.03 |
| Structure & packaging | pEa_SNUABM50_00159 | putative structural protein | *Serratia* phage BF | 100 | 98.45 |
| Nucleotide metabolism | pEa_SNUABM50_00162 | putative starvation-inducible DNA-binding protein | *Serratia* phage BF | 100 | 100 |
| Nucleotide metabolism | pEa_SNUABM50_00166 | putative GTP cyclohydrolase | *Serratia* phage BF | 100 | 100 |
| Nucleotide metabolism | pEa_SNUABM50_00168 | putative thymidine kinase | *Serratia* phage BF | 100 | 100 |
| Structure & packaging | pEa_SNUABM50_00169 | putative tail protein | *Serratia* phage BF | 100 | 100 |
| Structure & packaging | pEa_SNUABM50_00170 | putative membrane protein | *Serratia* phage BF | 100 | 100 |
| Structure & packaging | pEa_SNUABM50_00172 | putative membrane protein | *Yersinia* phage fHe-Yen9-03 | 100 | 72.73 |
| Nucleotide metabolism | pEa_SNUABM50_00174 | putative RNA ligase | *Serratia* phage BF | 100 | 99.28 |
| Structure & packaging | pEa_SNUABM50_00178 | putative membrane protein | *Serratia* phage BF | 100 | 97.83 |
| Nucleotide metabolism | pEa_SNUABM50_00180 | putative lysozyme | *Serratia* phage BF | 100 | 100 |
| Nucleotide metabolism | pEa_SNUABM50_00181 | putative sigma 54 modulation protein/ribosomal protein | *Serratia* phage BF | 100 | 100 |
| tRNA related | pEa_SNUABM50_00184 | putative tRNA nucleotidyl transferase | *Serratia* phage BF | 100 | 100 |
| Nucleotide metabolism | pEa_SNUABM50_00185 | putative nudix hydrolase | *Serratia* phage BF | 100 | 100 |
| Nucleotide metabolism | pEa_SNUABM50_00186 | putative glutaredoxin | *Serratia* phage BF | 100 | 100 |
| tRNA related | pEa_SNUABM50_00187 | putative aspartyl-tRNA amidotransferase | *Serratia* phage BF | 100 | 99.26 |
| Nucleotide metabolism | pEa_SNUABM50_00190 | putative Appr-1-p processing enzyme | *Serratia* phage BF | 100 | 100 |
| Structure & packaging | pEa_SNUABM50_00193 | putative neck protein | *Serratia* phage BF | 100 | 100 |
| Nucleotide metabolism | pEa_SNUABM50_00195 | putative deoxynucleotide monophosphate kinase | *Serratia* phage BF | 100 | 99.12 |
| Structure & packaging | pEa_SNUABM50_00196 | putative tail sheath protein | *Serratia* phage BF | 100 | 99.89 |
| Structure & packaging | pEa_SNUABM50_00197 | putative structural protein | *Yersinia* phage fHe-Yen9-04 | 100 | 93.45 |
| Structure & packaging | pEa_SNUABM50_00198 | putative structural protein | *Serratia* phage BF | 100 | 100 |
| Structure & packaging | pEa_SNUABM50_00199 | putative structural protein | *Serratia* phage BF | 100 | 100 |
| Structure & packaging | pEa_SNUABM50_00200 | putative head completion protein | *Serratia* phage BF | 100 | 100 |
| Structure & packaging | pEa_SNUABM50_00202 | putative structural protein | *Serratia* phage BF | 100 | 99.45 |
| Structure & packaging | pEa_SNUABM50_00203 | putative structural protein | *Serratia* phage BF | 100 | 99.46 |
| Structure & packaging | pEa_SNUABM50_00204 | putative structural protein | *Serratia* phage BF | 100 | 100 |
| Structure & packaging | pEa_SNUABM50_00205 | putative structural protein | *Serratia* phage BF | 100 | 100 |
| Structure & packaging | pEa_SNUABM50_00206 | putative structural protein | *Serratia* phage BF | 100 | 100 |
| Nucleotide metabolism | pEa_SNUABM50_00208 | putative ATPase | *Serratia* phage BF | 100 | 100 |
| Nucleotide metabolism | pEa_SNUABM50_00215 | putative thymidylate synthase | *Serratia* phage BF | 100 | 100 |
| Structure & packaging | pEa_SNUABM50_00216 | putative structural protein | *Serratia* phage BF | 100 | 99.85 |
| Structure & packaging | pEa_SNUABM50_00220 | putative structural protein | *Serratia* phage BF | 100 | 100 |
| Structure & packaging | pEa_SNUABM50_00221 | putative long tail fiber proximal subunit | *Serratia* phage BF | 100 | 99.46 |
| Structure & packaging | pEa_SNUABM50_00222 | putative structural protein | *Serratia* phage BF | 100 | 100 |
| Structure & packaging | pEa_SNUABM50_00223 | putative structural protein | *Serratia* phage BF | 100 | 100 |
| Structure & packaging | pEa_SNUABM50_00224 | putative structural protein | *Serratia* phage BF | 100 | 100 |
| Structure & packaging | pEa_SNUABM50_00225 | putative structural protein | *Serratia* phage BF | 100 | 100 |
| Structure & packaging | pEa_SNUABM50_00226 | putative structural protein | *Serratia* phage BF | 100 | 100 |
| Nucleotide metabolism | pEa_SNUABM50_00227 | putative NUDIX hydrolase family protein | *Serratia* phage BF | 100 | 100 |
| Structure & packaging | pEa_SNUABM50_00228 | putative tail sheath stabilizer and completion protein | *Serratia* phage BF | 100 | 100 |
| Structure & packaging | pEa_SNUABM50_00229 | putative structural protein | *Serratia* phage BF | 100 | 100 |
| Structure & packaging | pEa_SNUABM50_00230 | putative ATP-dependent Clp protease ATP-binding subunit clpA | *Serratia* phage BF | 100 | 99.74 |
| Structure & packaging | pEa_SNUABM50_00231 | putative structural protein | *Serratia* phage BF | 100 | 99.85 |
| Structure & packaging | pEa_SNUABM50_00232 | putative baseplate wedge | *Serratia* phage BF | 100 | 100 |
| Structure & packaging | pEa_SNUABM50_00233 | putative baseplate protein | *Serratia* phage BF | 100 | 100 |
| Lysis | pEa_SNUABM50_00234 | putative baseplate hub subunit and tail lysozyme | *Serratia* phage BF | 100 | 100 |
| Lysis | pEa_SNUABM50_00235 | putative T4-like phage baseplate hub and tail lysozyme | *Serratia* phage BF | 100 | 100 |
| Structure & packaging | pEa_SNUABM50_00236 | putative structural protein | *Serratia* phage BF | 100 | 99.87 |
| Structure & packaging | pEa_SNUABM50_00237 | putative baseplate wedge protein | *Serratia* phage BF | 100 | 100 |
| Structure & packaging | pEa_SNUABM50_00238 | putative structural protein | *Serratia* phage BF | 100 | 100 |
| Nucleotide metabolism | pEa_SNUABM50_00239 | putative RNA sigma factor for late transcription | *Serratia* phage BF | 100 | 100 |
| Nucleotide metabolism | pEa_SNUABM50_00240 | putative endonuclease subunit | *Serratia* phage BF | 100 | 100 |
| Nucleotide metabolism | pEa_SNUABM50_00241 | putative endonuclease subunit | *Serratia* phage BF | 100 | 99.72 |
| Structure & packaging | pEa_SNUABM50_00242 | putative EndoVII packaging and recombination endonuclease | *Serratia* phage BF | 100 | 99.38 |
| Structure & packaging | pEa_SNUABM50_00244 | putative baseplate hub subunit | *Serratia* phage BF | 100 | 100 |
| Structure & packaging | pEa_SNUABM50_00245 | putative tape measure protein | *Serratia* phage BF | 100 | 100 |
| Structure & packaging | pEa_SNUABM50_00246 | putative portal vertex protein | *Serratia* phage BF | 100 | 100 |
| Structure & packaging | pEa_SNUABM50_00248 | putative structural protein | *Serratia* phage BF | 100 | 100 |
| Structure & packaging | pEa_SNUABM50_00249 | putative prohead core protein | *Serratia* phage BF | 100 | 100 |
| Structure & packaging | pEa_SNUABM50_00250 | putative scaffolding protein | *Serratia* phage BF | 100 | 100 |
| Structure & packaging | pEa_SNUABM50_00251 | putative major capsid protein | *Serratia* phage BF | 100 | 100 |
| Nucleotide metabolism | pEa_SNUABM50_00253 | putative GIY-YIG nuclease family protein | *Serratia* phage BF | 100 | 100 |
| Structure & packaging | pEa_SNUABM50_00254 | putative tail fiber protein | *Serratia* phage BF | 100 | 100 |
| Nucleotide metabolism | pEa_SNUABM50_00257 | putative DNA polymerase | *Serratia* phage BF | 100 | 99.9 |
| Additional function | pEa_SNUABM50_00260 | putative serine/threonine protein phosphatase | *Serratia* phage BF | 100 | 100 |
| Additional function | pEa_SNUABM50_00261 | putative type I antifreeze protein | *Serratia* phage BF | 100 | 100 |
| Structure & packaging | pEa_SNUABM50_00262 | putative co-chaperonin GroES | *Serratia* phage BF | 100 | 100 |
| Structure & packaging | pEa_SNUABM50_00263 | putative structural protein | *Serratia* phage BF | 100 | 99.59 |
| Structure & packaging | pEa_SNUABM50_00264 | putative structural protein | *Serratia* phage BF | 100 | 100 |
| Structure & packaging | pEa_SNUABM50_00265 | putative structural protein | *Serratia* phage BF | 100 | 100 |
| Nucleotide metabolism | pEa_SNUABM50_00266 | putative RNaseH ribonuclease | *Serratia* phage BF | 100 | 100 |
| Structure & packaging | pEa_SNUABM50_00268 | putative terminase like protein | *Serratia* phage BF | 100 | 99.58 |
| Structure & packaging | pEa_SNUABM50_00269 | putative terminase large subunit | *Serratia* phage BF | 100 | 100 |
| Structure & packaging | pEa_SNUABM50_00270 | putative structural protein | *Serratia* phage BF | 100 | 100 |
| Nucleotide metabolism | pEa_SNUABM50_00271 | putative ssDNA binding protein | *Serratia* phage BF | 100 | 100 |
| Nucleotide metabolism | pEa_SNUABM50_00272 | putative UvsX protein | *Serratia* phage BF | 100 | 100 |
| Nucleotide metabolism | pEa_SNUABM50_00273 | putative UvsY portein | *Serratia* phage BF | 100 | 100 |
| Nucleotide metabolism | pEa_SNUABM50_00274 | putative DNA polymerase III epsilon subunit | *Serratia* phage BF | 100 | 100 |
| Nucleotide metabolism | pEa_SNUABM50_00275 | putative RNA-DNA + DNA-DNA helicase | *Serratia* phage BF | 100 | 100 |
| Structure & packaging | pEa_SNUABM50_00280 | putative structural protein | *Serratia* phage BF | 100 | 100 |
| Structure & packaging | pEa_SNUABM50_00281 | putative membrane protein | *Serratia* phage BF | 100 | 100 |
| Structure & packaging | pEa_SNUABM50_00283 | putative structural protein | *Serratia* phage BF | 100 | 100 |
| Nucleotide metabolism | pEa_SNUABM50_00284 | putative DNA primase subunit | *Serratia* phage BF | 100 | 99.71 |
| Nucleotide metabolism | pEa_SNUABM50_00285 | putative DNA primase-helicase | *Serratia* phage BF | 100 | 100 |
| Structure & packaging | pEa_SNUABM50_00289 | putative structural protein | *Serratia* phage BF | 100 | 99.67 |
| Structure & packaging | pEa_SNUABM50_00290 | putative structural protein | *Serratia* phage BF | 100 | 100 |
| Structure & packaging | pEa_SNUABM50_00292 | putative structural protein | *Serratia* phage BF | 100 | 99.54 |
| Structure & packaging | pEa_SNUABM50_00293 | putative structural protein | *Serratia* phage BF | 100 | 100 |
| Nucleotide metabolism | pEa_SNUABM50_00294 | putative restriction endonuclease type II like-protein | *Serratia* phage BF | 100 | 100 |
| Structure & packaging | pEa_SNUABM50_00296 | putative structural protein | *Serratia* phage BF | 100 | 100 |
| Nucleotide metabolism | pEa_SNUABM50_00297 | putative aerobic ribonucleotide-diphosphate reductase alpha subunit | *Serratia* phage BF | 100 | 99.87 |
| Nucleotide metabolism | pEa_SNUABM50_00298 | putative aerobic ribonucleotide-diphosphate reductase beta subunit | *Serratia* phage BF | 100 | 100 |
| Structure & packaging | pEa_SNUABM50_00299 | putative membrane protein | *Serratia* phage BF | 100 | 100 |
| Structure & packaging | pEa_SNUABM50_00301 | putative structural protein | *Serratia* phage BF | 100 | 100 |
| Nucleotide metabolism | pEa_SNUABM50_00302 | putative nucleotide pyrophosphohydrolase | *Serratia* phage BF | 100 | 99.57 |
| Structure & packaging | pEa_SNUABM50_00304 | putative structural protein | *Serratia* phage BF | 100 | 100 |
| Structure & packaging | pEa_SNUABM50_00305 | putative membrane protein | *Serratia* phage BF | 100 | 100 |
| Nucleotide metabolism | pEa_SNUABM50_00306 | putative dihydrofolate reductase | *Serratia* phage BF | 100 | 99.43 |
| Nucleotide metabolism | pEa_SNUABM50_00307 | putative ribonuclease H | *Serratia* phage BF | 100 | 100 |
| Nucleotide metabolism | pEa_SNUABM50_00308 | putative DNA helicase Dda | *Serratia* phage BF | 100 | 100 |
| Structure & packaging | pEa_SNUABM50_00310 | putative structural protein | *Serratia* phage BF | 100 | 98.89 |
| Nucleotide metabolism | pEa_SNUABM50_00312 | putative translation initiation factor IF-3 | *Serratia* phage BF | 100 | 100 |
| Structure & packaging | pEa_SNUABM50_00313 | putative ATP-dependent Clp protease | *Serratia* phage BF | 100 | 100 |
| Nucleotide metabolism | pEa_SNUABM50_00314 | putative DnaJ-like protein | *Serratia* phage BF | 100 | 100 |
| Structure & packaging | pEa_SNUABM50_00315 | putative structural protein | *Serratia* phage BF | 100 | 100 |
| Structure & packaging | pEa_SNUABM50_00316 | putative structural protein | *Serratia* phage BF | 100 | 99.88 |
| Structure & packaging | pEa_SNUABM50_00317 | putative membrane protein | *Serratia* phage BF | 100 | 100 |
| Nucleotide metabolism | pEa_SNUABM50_00318 | putative topoisomerase II large subunit | *Serratia* phage BF | 100 | 100 |
| Nucleotide metabolism | pEa_SNUABM50_00319 | putative DNA topoisomerase II medium subunit | *Serratia* phage BF | 100 | 99.79 |
| Structure & packaging | pEa_SNUABM50_00320 | putative structural protein | *Serratia* phage BF | 100 | 99.9 |
| Nucleotide metabolism | pEa_SNUABM50_00322 | putative DNA polymerase III alpha subunit | *Serratia* phage BF | 100 | 99.44 |
| Structure & packaging | pEa_SNUABM50_00324 | putative co-chaperonin GroES | *Serratia* phage BF | 100 | 100 |
| Nucleotide metabolism | pEa_SNUABM50_00326 | putative sliding clamp loader subunit | *Serratia* phage BF | 100 | 100 |
| Additional function | pEa_SNUABM50_00327 | putative phosphoglycolate phosphatase | *Serratia* phage BF | 100 | 100 |
| Structure & packaging | pEa_SNUABM50_00328 | putaive structural protein | *Serratia* phage BF | 100 | 100 |
| Structure & packaging | pEa_SNUABM50_00329 | putaive structural protein | *Serratia* phage BF | 100 | 100 |
| Additional function | pEa_SNUABM50_00334 | putative TelA like protein | *Serratia* phage BF | 100 | 99.74 |
| Nucleotide metabolism | pEa_SNUABM50_00335 | putative nucleotide reductase subunit C | *Serratia* phage BF | 100 | 100 |
| Additional function | pEa_SNUABM50_00336 | putative metallopeptidase | *Serratia* phage BF | 100 | 100 |
| Structure & packaging | pEa_SNUABM50_00337 | putative membrane protein | *Serratia* phage BF | 100 | 99.45 |
| Structure & packaging | pEa_SNUABM50_00338 | putative structural protein | *Serratia* phage BF | 100 | 100 |
| Structure & packaging | pEa_SNUABM50_00342 | putative membrane protein | *Serratia* phage BF | 100 | 100 |
| Nucleotide metabolism | pEa_SNUABM50_00345 | putative ADP-ribosylglycohydrolase | *Serratia* phage BF | 100 | 100 |
| Nucleotide metabolism | pEa_SNUABM50_00355 | putative nicotinamide nucleotide adenylyltransferase | *Serratia* phage BF | 100 | 99.72 |
| Nucleotide metabolism | pEa_SNUABM50_00356 | putative nicotinamide mononucleotide transporter PnuC | *Serratia* phage BF | 100 | 100 |
| Structure & packaging | pEa_SNUABM50_00363 | putative structural protein | *Serratia* phage BF | 100 | 100 |
| tRNA | pEa_SNUABM50_00367 | tRNA-Ser |  |  |  |
| Nucleotide metabolism | pEa_SNUABM50_00372 | putative ATPase | *Serratia* phage BF | 100 | 98.98 |
| tRNA | pEa_SNUABM50_00373 | tRNA-Trp |  |  |  |
| Nucleotide metabolism | pEa_SNUABM50_00375 | putative nucleotidase | *Serratia* phage BF | 100 | 100 |
| tRNA | pEa_SNUABM50_00378 | tRNA-Thr |  |  |  |
| tRNA related | pEa_SNUABM50_00390 | putative peptidyl-tRNA hydrolase | *Serratia* phage BF | 100 | 100 |
| tRNA | pEa_SNUABM50_00391 | tRNA-Leu |  |  |  |
| Structure & packaging | pEa_SNUABM50_00392 | putative membrane protein | *Serratia* phage BF | 100 | 100 |
| tRNA | pEa_SNUABM50_00410 | tRNA-Arg |  |  |  |
| tRNA | pEa_SNUABM50_00416 | tRNA-Pyl |  |  |  |
| tRNA | pEa_SNUABM50_00418 | tRNA-Met |  |  |  |
| tRNA | pEa_SNUABM50_00424 | tRNA-Leu |  |  |  |
| tRNA | pEa_SNUABM50_00437 | tRNA-Phe |  |  |  |
| tRNA | pEa_SNUABM50_00442 | tRNA-Lys |  |  |  |
| Structure & packaging | pEa_SNUABM50_00443 | putative HNH endonuclease | *Yersinia* phage fHe-Yen9-04 | 99 | 65.52 |
| Structure & packaging | pEa_SNUABM50_00447 | putative membrane protein | *Serratia* phage BF | 100 | 99.12 |
| tRNA | pEa_SNUABM50_00448 | tRNA-Leu |  |  |  |
| Structure & packaging | pEa_SNUABM50_00451 | putative membrane protein | *Serratia* phage BF | 100 | 100 |
| Nucleotide metabolism | pEa_SNUABM50_00453 | putative AAA family ATPase | *Serratia* phage BF | 100 | 100 |
| tRNA | pEa_SNUABM50_00455 | tRNA-Glu |  |  |  |
| tRNA | pEa_SNUABM50_00456 | tRNA-Ser |  |  |  |
| tRNA | pEa_SNUABM50_00458 | tRNA-Ser |  |  |  |
| tRNA | pEa_SNUABM50_00459 | tRNA-Ser |  |  |  |
| Structure & packaging | pEa_SNUABM50_00461 | putative ATP-dependent Clp protease proteolytic subunit | *Serratia* phage BF | 100 | 100 |
| tRNA | pEa_SNUABM50_00464 | tRNA-Ile |  |  |  |
| tRNA | pEa_SNUABM50_00468 | tRNA-Asn |  |  |  |
| tRNA | pEa_SNUABM50_00469 | tRNA-Gln |  |  |  |
| tRNA | pEa_SNUABM50_00470 | tRNA-Gly |  |  |  |
| tRNA | pEa_SNUABM50_00475 | tRNA-Asp |  |  |  |
| tRNA | pEa_SNUABM50_00477 | tRNA-Arg |  |  |  |
| tRNA | pEa_SNUABM50_00482 | tRNA-Pro |  |  |  |
| tRNA | pEa_SNUABM50_00483 | tRNA-Pro |  |  |  |
| tRNA | pEa_SNUABM50_00484 | tRNA-Pro |  |  |  |
| tRNA | pEa_SNUABM50_00485 | tRNA-Val |  |  |  |
| tRNA | pEa_SNUABM50_00488 | tRNA-His |  |  |  |
| tRNA | pEa_SNUABM50_00489 | tRNA-Phe |  |  |  |
| tRNA | pEa_SNUABM50_00491 | tRNA-Lys |  |  |  |
| Structure & packaging | pEa_SNUABM50_00495 | putative membrane protein | *Serratia* phage BF | 100 | 98.13 |
| tRNA | pEa_SNUABM50_00499 | tRNA-Tyr |  |  |  |
| tRNA | pEa_SNUABM50_00504 | tRNA-Cys |  |  |  |
| tRNA | pEa_SNUABM50_00505 | tRNA-Lys |  |  |  |
| tRNA | pEa_SNUABM50_00507 | tRNA-Met |  |  |  |
| tRNA | pEa_SNUABM50_00508 | tRNA-Met |  |  |  |
| tRNA | pEa_SNUABM50_00509 | tRNA-Ala |  |  |  |
| Structure & packaging | pEa_SNUABM50_00510 | putative structural protein | *Serratia* phage BF | 100 | 97.48 |
| Nucleotide metabolism | pEa_SNUABM50_00511 | putative S-adenosyl-L- methionine-dependent methyltransferase | *Serratia* phage BF | 100 | 100 |
| Structure & packaging | pEa_SNUABM50_00512 | putative structural protein | *Serratia* phage BF | 100 | 99.47 |
| Structure & packaging | pEa_SNUABM50_00518 | putative membrane protein | *Serratia* phage BF | 100 | 100 |
| Structure & packaging | pEa_SNUABM50_00519 | putative membrane protein | *Serratia* phage BF | 100 | 99.3 |
| Nucleotide metabolism | pEa_SNUABM50_00531 | putative subfamily RNA polymerase sigma-70 subunit | *Serratia* phage BF | 100 | 99.7 |
| Structure & packaging | pEa_SNUABM50_00534 | putative structural protein | *Serratia* phage BF | 100 | 100 |
| Structure & packaging | pEa_SNUABM50_00535 | putative membrane protein | *Serratia* phage BF | 100 | 98.61 |
| Additional function | pEa_SNUABM50_00539 | putative C4-type zinc finger domain-containing protein | *Serratia* phage BF | 100 | 100 |
| Structure & packaging | pEa_SNUABM50_00547 | putative HNH endonuclease | *Serratia* phage BF | 100 | 100 |
| Structure & packaging | pEa_SNUABM50_00553 | putative HNH endonuclease | *Serratia* phage BF | 100 | 99.65 |
| Nucleotide metabolism | pEa_SNUABM50_00554 | putative thioredoxin | *Serratia* phage BF | 100 | 100 |
| Structure & packaging | pEa_SNUABM50_00555 | putative membrane protein | *Serratia* phage BF | 100 | 100 |
| Structure & packaging | pEa_SNUABM50_00557 | putative membrane protein | *Serratia* phage BF | 100 | 100 |
| Structure & packaging | pEa_SNUABM50_00558 | putative membrane protein | *Serratia* phage BF | 100 | 99.17 |
| Structure & packaging | pEa_SNUABM50_00559 | putative membrane protein | *Serratia* phage BF | 100 | 100 |
| Structure & packaging | pEa_SNUABM50_00560 | putative membrane protein | *Serratia* phage BF | 100 | 99.09 |
| Structure & packaging | pEa_SNUABM50_00561 | putative membrane protein | *Serratia* phage BF | 100 | 100 |
| Structure & packaging | pEa_SNUABM50_00562 | putative membrane protein | *Serratia* phage BF | 100 | 99.53 |
| Additional function | pEa_SNUABM50_00565 | putative PE-PGRS family protein | *Serratia* phage BF | 100 | 98.93 |
| Nucleotide metabolism | pEa_SNUABM50_00566 | putative DNA condensation protein | *Serratia* phage BF | 100 | 96.26 |
| Nucleotide metabolism | pEa_SNUABM50_00570 | putative DNA condensation protein | *Serratia* phage BF | 100 | 98.18 |
| Nucleotide metabolism | pEa_SNUABM50_00571 | putative DNA condensation protein | *Serratia* phage BF | 100 | 100 |
| Structure & packaging | pEa_SNUABM50_00573 | putative structural protein | *Serratia* phage BF | 100 | 99.55 |
| Nucleotide metabolism | pEa_SNUABM50_00574 | putative DNA condensation protein | *Serratia* phage BF | 100 | 99.72 |
