# Supplementary material for: Characterization of Novel Erwinia amylovora Jumbo Bacteriophages from Eneladusvirus Genus
Source: Viruses. 2020 Nov 30;12(12):1373. doi: 10.3390/v12121373 (PMC7760394; doi:10.3390/v12121373)
Supplement: Supplementary file 1 [file viruses-12-01373-s001.zip › Supplementary Table S5.docx]

**Table S6. Characteristics of the LC-MS/MS detected proteins of the *E. amylovora* phages pEa_SNUABM_12, pEa_SNUABM_47, and pEa_SNUABM_50.**

| **#** | **Putative function** | **Molecular weight (kDa)** | **Number of identified peptides** | | | **% coverage** | | |
| --- | --- | --- | --- | --- | --- | --- | --- | --- |
|  |  |  | **pEa_12** | **pEa_47** | **pEa_50** | **pEa_12** | **pEa_47** | **pEa_50** |
| 1 | hypothetical protein | 381.1 | 27 | 56 | 197 | 7 | 12 | 27 |
| 2 | putative long tail fiber proximal subunit | 147 | 46 | 33 | 123 | 13 | 8 | 14 |
| 3 | putative baseplate wedge | 131.6 | 18 | 36 | 100 | 11 | 15 | 35 |
| 4 | putative structural protein | 112.3 | 3 | 13 | 53 | 5 | 10 | 29 |
| 5 | hypothetical protein | 99.7 | 3 | 7 | 40 | 2 | 7 | 29 |
| 6 | hypothetical protein | 99 | 6 | 8 | 31 | 8 | 8 | 22 |
| 7 | hypothetical protein | 97.3 | 6 | 8 | 25 | 5 | 7 | 20 |
| 8 | putative lysozyme | 96.3 | 5 | 6 | 49 | 5 | 7 | 30 |
| 9 | putative tail sheath protein | 95.8 | 73 | 100 | 249 | 28 | 36 | 49 |
| 10 | putative structural protein | 92.8 | 10 | 6 | 44 | 10 | 6 | 22 |
| 11 | putative structural protein | 88.4 | 5 | 7 | 30 | 5 | 9 | 22 |
| 12 | putative structural protein | 78.6 | 6 | 7 | 49 | 10 | 10 | 48 |
| 13 | putative structural protein | 68.5 | 12 | 14 | 42 | 11 | 15 | 34 |
| 14 | putative portal vertex protein | 64.4 | 5 | 8 | 30 | 6 | 8 | 27 |
| 15 | putative structural protein | 60.7 | 2 | 1 | 11 | 4 | 2 | 13 |
| 16 | hypothetical protein | 59 | 4 | 10 | 30 | 8 | 17 | 26 |
| 17 | putative tail fiber protein | 58.8 | 13 | 14 | 29 | 16 | 21 | 24 |
| 18 | putative structural protein | 56.6 | 4 |  | 18 | 6 |  | 25 |
| 19 | putative tail sheath stabilizer and completion protein | 51.2 |  | 5 | 18 |  | 10 | 28 |
| 20 | structural protein | 48.7 | 8 | 17 |  | 24 | 24 |  |
| 21 | hypothetical protein | 42.7 | 2 |  | 21 | 7 |  | 29 |
| 22 | putative scaffolding protein | 42.6 |  | 7 | 12 |  | 13 | 19 |
| 23 | putative precursor of major capsid protein/ putative major capsid protein | 42.1 | 834 | 1267 | 1337 | 72 | 76 | 76 |
| 24 | hypothetical protein | 41.6 |  | 21 | 24 |  | 32 | 30 |
| 25 | putative DNA condensation protein | 41.6 | 11 | 8 | 9 | 17 | 17 | 19 |
| 26 | putative DNA condensation protein | 41.5 | 9 | 20 | 25 | 17 | 25 | 29 |
| 27 | structural protein | 41 | 20 | 27 | 43 | 18 | 20 | 20 |
| 28 | hypothetical protein | 40.8 | 6 | 16 | 15 | 12 | 24 | 27 |
| 29 | putative structural protein | 40.2 | 44 | 76 | 100 | 45 | 48 | 45 |
| 30 | putative structural protein | 40.2 | 22 | 50 | 52 | 42 | 68 | 70 |
| 31 | putative DNA condensation protein | 40.2 | 10 | 21 | 20 | 25 | 38 | 36 |
| 32 | putative structural protein | 39.7 | 17 | 31 | 46 | 49 | 62 | 73 |
| 33 | putative DNA condensation protein | 38 | 3 | 5 | 6 | 6 | 11 | 11 |
| 34 | putative structural protein | 36.5 | 17 | 33 | 47 | 44 | 58 | 59 |
| 35 | hypothetical protein | 35.2 |  | 7 | 12 |  | 9 | 10 |
| 36 | putative structural protein | 35.1 | 17 | 32 | 38 | 16 | 24 | 24 |
| 37 | putative structural protein | 33.5 |  | 1 | 5 |  | 11 | 16 |
| 38 | putative structural protein | 32 | 24 | 19 | 40 | 18 | 14 | 18 |
| 39 | SPFH domain containing protein | 30.6 | 8 | 15 | 12 | 23 | 38 | 23 |
| 40 | Ig domain containing protein | 30 | 115 | 216 | 402 | 56 | 59 | 60 |
| 41 | putative structural protein | 29.7 | 18 | 32 | 57 | 60 | 60 | 70 |
| 42 | putative neck protein | 29.5 | 5 | 25 | 40 | 28 | 59 | 72 |
| 43 | putative structural protein | 28.7 |  | 9 | 19 |  | 32 | 37 |
| 44 | putative structural protein | 27.8 | 7 | 19 | 29 | 20 | 62 | 65 |
| 45 | putative PE-PGRS family protein | 25.9 | 1 | 6 | 13 | 10 | 18 | 28 |
| 46 | putative structural protein | 25.8 | 20 | 15 | 23 | 29 | 18 | 29 |
| 47 | hypothetical protein | 25.2 | 13 | 17 | 12 | 27 | 37 | 35 |
| 48 | hypothetical protein | 25 | 1 | 1 | 3 | 6 | 6 | 4 |
| 49 | hypothetical protein | 24.3 | 19 | 18 | 46 | 50 | 40 | 67 |
| 50 | hypothetical protein | 24.3 | 5 | 4 | 8 | 18 | 11 | 18 |
| 51 | putative structural protein | 24.1 | 19 | 28 | 40 | 30 | 36 | 36 |
| 52 | putative prohead core scaffolding protein | 23.2 |  |  | 1 |  |  | 4 |
| 53 | hypothetical protein | 22.7 | 7 | 5 | 16 | 15 | 17 | 35 |
| 54 | putative structural protein | 22.5 | 109 | 324 | 467 | 57 | 69 | 94 |
| 55 | putative structural protein | 22.1 | 7 | 7 | 17 | 25 | 31 | 32 |
| 56 | hypothetical protein | 22.1 |  | 1 | 2 |  | 8 | 13 |
| 57 | putative structural protein | 21.8 | 3 | 5 | 10 | 7 | 13 | 14 |
| 58 | putative structural protein | 21.1 | 34 | 48 | 77 | 57 | 71 | 71 |
| 59 | putative structural protein | 21 | 6 | 6 | 20 | 20 | 20 | 24 |
| 60 | hypothetical protein | 20.8 | 8 | 13 | 19 | 23 | 40 | 44 |
| 61 | putative DnaJ-like protein | 20.6 | 4 | 3 | 8 | 15 | 23 | 42 |
| 62 | structural protein | 20.1 | 15 | 16 | 36 | 25 | 25 | 25 |
| 63 | hypothetical protein | 20.1 | 7 | 8 | 10 | 23 | 13 | 33 |
| 64 | structural protein | 19.8 | 7 | 10 | 27 | 31 | 16 | 32 |
| 65 | hypothetical protein | 19.1 | 34 | 36 | 49 | 9 | 9 | 9 |
| 66 | putative structural protein | 19 | 64 | 88 | 136 | 35 | 45 | 45 |
| 67 | putative structural protein | 19 | 8 | 11 | 10 | 13 | 39 | 41 |
| 68 | hypothetical protein | 18.4 | 4 | 6 | 8 | 9 | 9 | 9 |
| 69 | putative baseplate hub subunit and tail lysozyme | 18.1 | 1 | 2 | 1 | 9 | 9 | 9 |
| 70 | putative structural protein | 18 | 20 | 29 | 35 | 54 | 60 | 60 |
| 71 | putative structural protein | 17 | 8 | 16 | 11 | 15 | 19 | 16 |
| 72 | hypothetical protein | 16.6 | 6 | 5 | 7 | 28 | 34 | 31 |
| 73 | putative acyl carrier protein | 15.6 | 4 | 3 | 5 | 8 | 8 | 15 |
| 74 | putative baseplate protein | 14.8 | 7 | 6 | 12 | 23 | 16 | 16 |
| 75 | hypothetical protein | 13.7 |  |  | 1 |  |  | 12 |
| 76 | hypothetical protein | 13.6 | 1 | 8 | 7 | 9 | 20 | 20 |
| 77 | putative structural protein | 13.6 | 1 | 2 | 3 | 9 | 9 | 18 |
| 78 | hypothetical protein | 13.2 |  | 1 | 1 |  | 14 | 13 |
| 79 | putative structural protein | 12.2 | 1 | 3 | 4 | 12 | 24 | 33 |
| 80 | putative structural protein | 12.2 |  |  | 2 |  |  | 25 |
| 81 | hypothetical protein | 12.1 | 1 | 3 |  | 14 | 14 |  |
| 82 | hypothetical protein | 11.3 |  | 3 | 2 |  | 35 | 18 |
| 83 | hypothetical protein | 10.9 | 2 | 2 | 4 | 12 | 12 | 39 |
| 84 | hypothetical protein | 10.5 | 3 | 15 | 12 | 34 | 52 | 43 |
| 85 | hypothetical protein | 10.4 | 2 | 3 | 6 | 11 | 29 | 29 |
| 86 | hypothetical protein | 9.4 | 2 | 10 | 9 | 26 | 53 | 53 |
| 87 | hypothetical protein | 8.8 | 1 | 5 | 8 | 14 | 43 | 43 |
| 88 | hypothetical protein | 8.7 | 4 | 1 | 3 | 28 | 15 | 28 |
| 89 | putative structural protein | 8.4 | 4 | 7 | 12 | 67 | 75 | 75 |
